# Exploring the thermodynamic criteria for responsive adsorption processes†
†Electronic supplementary information (ESI) available. See DOI: 10.1039/c9sc01299k


**DOI:** 10.1039/c9sc01299k

**Published:** 2019-04-15

**Authors:** Jack D. Evans, Simon Krause, Stefan Kaskel, Martin B. Sweatman, Lev Sarkisov

**Affiliations:** a Department of Inorganic Chemistry , Technische Universität Dresden , Bergstraße 66 , 01062 Dresden , Germany . Email: jack.evans@tu-dresden.de; b School of Engineering , University of Edinburgh , Edinburgh EH9 3FB , UK

## Abstract

We describe a general model to explore responsive adsorption processes in flexible porous materials. This model combines mean field formalism of the osmotic potential, classical density functional theory of adsorption in slit pore models and generic potential functions which represent the Helmholtz free energy landscape of a porous system. Using this model, we focus on recreating flexible adsorption phenomena observed in prototypical metal–organic frameworks, especially the recently discovered effect of negative gas adsorption (NGA). We identify the key characteristics required for the model to generate unusual adsorption processes and subsequently employ an extensive parametric study to outline conditions under which gate-opening and NGA are observed. This powerful approach will guide the design of responsive porous materials and the discovery of entirely new adsorption processes.

## Abstract 2

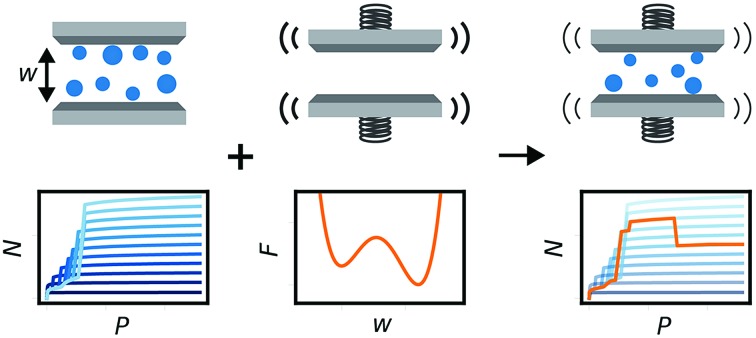
A general thermodynamic model to investigate responsive adsorption processes in flexible porous materials.

## Introduction

New families of porous crystals have captivated the efforts and imagination of researchers and find use in many important applications,^1^ including catalysis and gas separations.^2^,^3^ A particular class of these materials are soft porous crystals (SPCs), which incorporate a dynamic and adaptive pore network, changing in both size and shape.^4^ The flexibility exhibited by SPCs provides advanced functions such as responsive materials, where the pore network changes in response to external stimuli including mechanical force, electric fields, light or the presence of particular species.^5^,^6^ This unique property can be potentially exploited for a number of applications, including sensing, drug delivery and improved gas storage with thermal management.^7^–^9^

SPC materials that are responsive to the specific stimulus of gas adsorption typically exhibit one or more distinct steps in their gas adsorption isotherms, attributed to structural transformation of the host material.^10^ Examples of these responsive adsorption processes include gate-opening and breathing phenomena demonstrated by metal–organic framework (MOF) materials.^11^,^12^ Gate-opening refers to a particular (and sometimes subtle) change in the structure of the porous network at a specific adsorptive pressure.^13^ This change in the structure “opens” previously inaccessible pore volume to the adsorbing molecules producing a transition from low porosity narrow pore (np) state to more porous open pore (op) state, leading to a stepwise change in the adsorption isotherm. Recently, it was demonstrated that this effect can be exploited to maximize the deliverable amount of methane in storage applications.^9^ Breathing processes, however, begin with an open and high porosity conformation (op) at zero or low pressure of adsorbate. Upon adsorption, the structure spontaneously collapses into a less porous state (np). The deformation to a np state arises from the strong adsorption enthalpy attributed to smaller pore sizes.^14^ Subsequently, an increase in pressure and the amount adsorbed leads to a second transition back to the op configuration. The specific pressure of these transitions depends on the adsorbing gas and temperature.^15^ In many systems the op configuration is only a stable empty host configuration above a certain threshold temperature, which can result in the absence of the transitions detailed above.^16^ Coudert and coworkers developed a systematic description of the thermodynamics of these transitions, which has enabled the production of detailed phase diagrams and improved understand of coadsorption behavior.^14^,^17^,^18^ However, previous work has not considered a diverse assortment of host free energy profiles, which are representative of different SPCs.

Of particular interest in this work is the possibility for a significant energy barrier between op and np phases. This may result in the adsorption process populating metastable states and, upon transition to the np form, some of the adsorbate is actually released back to the gas phase, leading to negative gas adsorption (NGA).^19^ NGA phenomena were recently discovered for methane and *n*-butane adsorption in DUT-49.^20^ This has since spurred extensive experimental investigation to elucidate the effect of crystallite size and the collective nature of the process.^21^,^22^ Evans, Bocquet and Coudert also provided an initial theoretical description of NGA highlighting the ligand buckling motif that produces a large energy barrier between the op and np structures of DUT-49.^23^ Importantly, much of the investigation of NGA has centered on one material (DUT-49). However, in the present work we aim to demonstrate this counterintuitive phenomena in general models of porous materials.

Here we specifically develop a general, minimal theoretical model capable of reproducing responsive adsorption processes that include metastable transitions. The model is inspired by several recent studies on adsorption phenomena in flexible materials. Coudert and coworkers elaborated the thermodynamics of adsorption in flexible porous materials using the osmotic ensemble framework.^24^ In particular, they proposed a mean field simplification of the osmotic configurational partition function, which leads to an elegant separation of variables within the configurational integral—a simple formulation of the osmotic potential in terms of the free energy of the solid phase and the adsorption isotherm of fluid inside that phase. Ravikovitch and Neimark employed classical non-local density functional theory (NLDFT) to propose a model of elastic deformations of the solid matrix describing contraction or swelling upon adsorption.^25^ These ideas were later extended by Kowalczyk and coworkers to explain adsorption induced deformation in microporous carbons.^26^ While in this study they used Monte Carlo simulations of adsorption instead of NLDFT, a common approach begun to emerge where the flexible porous structure is treated as a slit pore with a spring attached to the walls. Several MOFs have a structure and deformation modes that can be adequately represented as a system of stacked layers separated by springs. In particular, Numaguchi and coworkers developed a theoretical description of structural transitions in ELM-11.^27^ Recently, Siderius and coworkers also used a combination of flat-histogram sampling Monte Carlo methods and slit pore models to study capillary phase transitions in deformable adsorbent materials.^28^ They proposed how these approaches can be used to construct a pore size distribution for materials with deformable structure.

**Fig. 1:**
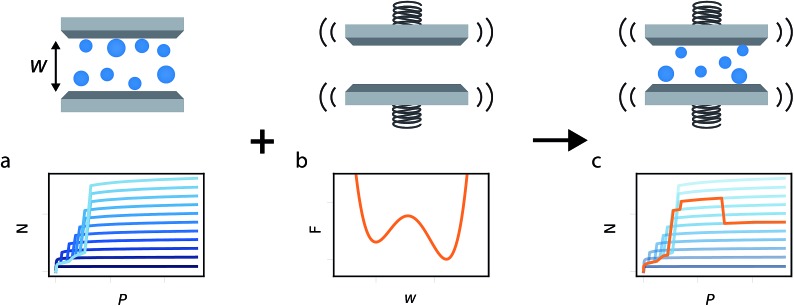
Illustration of the methodology used to chart representative gas adsorption isotherms of responsive porous materials. (a) A series of isotherms a different pore widths, identified by different shades of blue. (b) The free energy profile describing the pore width. (c) A responsive isotherm calculated from the series of isotherms, as the minimum of *Ω*_os_, and identified by an orange path.

In this study we build on all these previous developments to produce the general method illustrated in Fig. 1. Flexible porous materials are treated as a simple slit pore with a spring attached to its walls, and various functional forms are used to describe this spring potential. A density functional theory (DFT) description of adsorption in slit pore models provides a route for direct calculation of the grand potential of the adsorbed fluid in a pore of fixed width (a much more computationally efficient technique than Monte Carlo, for this purpose). The mean field formulation of the osmotic potential by Coudert *et al.* subsequently allows for the combination of grand potential from the DFT calculations with the Helmholtz free energy of the solid, represented by a potential. This simple model allows for the development of the minimum number of parameters required to formulate a new model capable of capturing the NGA effect. Furthermore, we investigate the general Helmholtz free energy profile of the host material and the external thermodynamic criteria required for responsive adsorption phenomena.

## Methodology

Thermodynamic equilibrium is the minimum of the osmotic potential (*Ω*_os_) and, as discussed previously, we adopt the mean field approach of Coudert *et al.*eqn (1), where *F*_host_ is the Helmholtz free energy of the porous host, *P* is gas pressure and *Ω*(*T*,*P*,*w*) is the grand potential of the adsorbed fluid.^24^

1 *Ω*_os_(*T*,*P*,*w*) = *F*_host_(*w*) + *PV* + *Ω*(*T*,*P*,*w*)

To generate a library of states (of different pore width) of the system and corresponding grand potential (*Ω*(*T*,*P*,*w*)) we use classical density functional theory (DFT) for simple fluids confined in slit pore structures.^29^ Slit pore models have been employed to study materials with a broad pore size distribution^26^ or in stacked-layer materials, such as ELM-11.^27^ While these models may prove difficult to provide quantitative agreement to experimental results of other porous materials they allow for the exploration of adsorption phenomena in a simplified system. Additionally, the described methodology can be readily improved by using a more complex kernel, if required.

We define the grand potential functional of the average one-body density *ρ*(*r*), eqn (2), where *F*[*ρ*(*r*)] is the intrinsic Helmholtz free-energy functional, *V*_ext_ is the external potential and *μ*(*ρ*_B_) is the reservoir fluid chemical potential, which is determined by the reservoir bulk density (*ρ*_B_).2
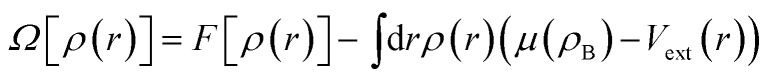



Several approaches to estimate these functionals have been developed^30^ and, in this work, we employ standard mean field DFT with a fundamental measure functional for the hard sphere term within the Helmholtz free energy functional *F*[*ρ*]. This provides a large kernel of adsorption isotherms for different slit pore width (*w*), including data of adsorbed amount and the grand potential, provided explicitly. The specific technical details of this model are provided in the ESI.†

This methodology allows for a direct comparison of the grand potential for a given pressure and temperature at different pore widths, demonstrated in Fig. S4.† Subsequently, the complete adsorption profile for a flexible pore is generated by following the minimum of *Ω*_os_ as a function of pressure.

In this study, we considered two athermal functions of Helmholtz free energy to describe the flexibility, or phases, of the slit pore systems of width *w*. This includes a harmonic-type potential for a system with a single minimum (eqn (3)) and a bistable-type potential (eqn (4)), which describes a system with two minima separated by barrier.3
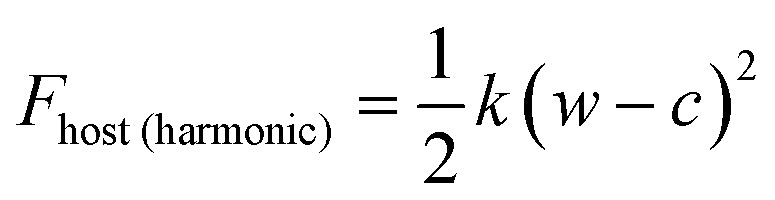

4 *F*_host(bistable)_ = *c*_1_(*w* – *c*_2_)^4^ – *c*_3_(*w* – *c*_4_)^2^ + *c*_5_(*w* – *c*_6_)

The variables (*k* and *c*) of eqn (3) are chosen to generate a potential surface centered on pore width (*w*_min_) with a given stiffness, or looseness, defined by (*k*). Similarly, the coefficients (*c*_*i*_) of eqn (4) are selected to produce a potential surface with minima at chosen width values (*w*_thin_, *w*_wide_), relative energetic stability (*E*_thin_, *E*_wide_) and barrier height, measured from the lowest energetic minimum (*E*_barrier_).

Importantly, gas adsorption does not only follow the minimum of *Ω*_os_. We impose an additional kinetic criteria, a threshold activation energy (*Ω*_crit_), to our model. As discussed by Numaguchi *et al.*, structural transitions should only occur when the activation energy (or energetic barrier between the current state and the *Ω*_os_ minimum state) becomes equal or less than a characteristic system energy fluctuation.^27^ The inclusion of this vital condition allows for the simulation of metastable transitions and also reflects the finite observation time of experimental adsorption. We set energy fluctuations of the system (*Ω*_crit_) to be 6*kT* and we note this is used here as a free parameter. The ESI† includes a detailed description of *Ω*_crit_, as it relates to these systems.

An example of the code used to generate the responsive isotherms can be found at the data repository of J. D. Evans at https://github.com/jackevansadl/supp-data. We encourage researchers to explore this methodology by combining alternative isotherm kernels and complex host free energy potentials.

The results, illustrated here, are displayed in reduced units (denoted by *) relative to the length of the Lennard-Jones diameter of the adsorbate fluid particle (*σ*_ff_) and fluid–fluid interaction strength (*ε*_ff_). For example, temperature in reduced units is given as 
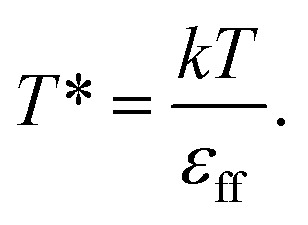
 A full description of the reduced units is summarized in the ESI (Table S1†), including how they can be mapped onto a corresponding atomistic system. Furthermore, in this study adsorption isotherms are presented as adsorbed density (N*) as a function of configurational activity (λ*), which is calculated from the chemical potential as 
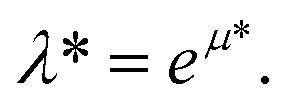


## Results and discussion

**Fig. 2:**
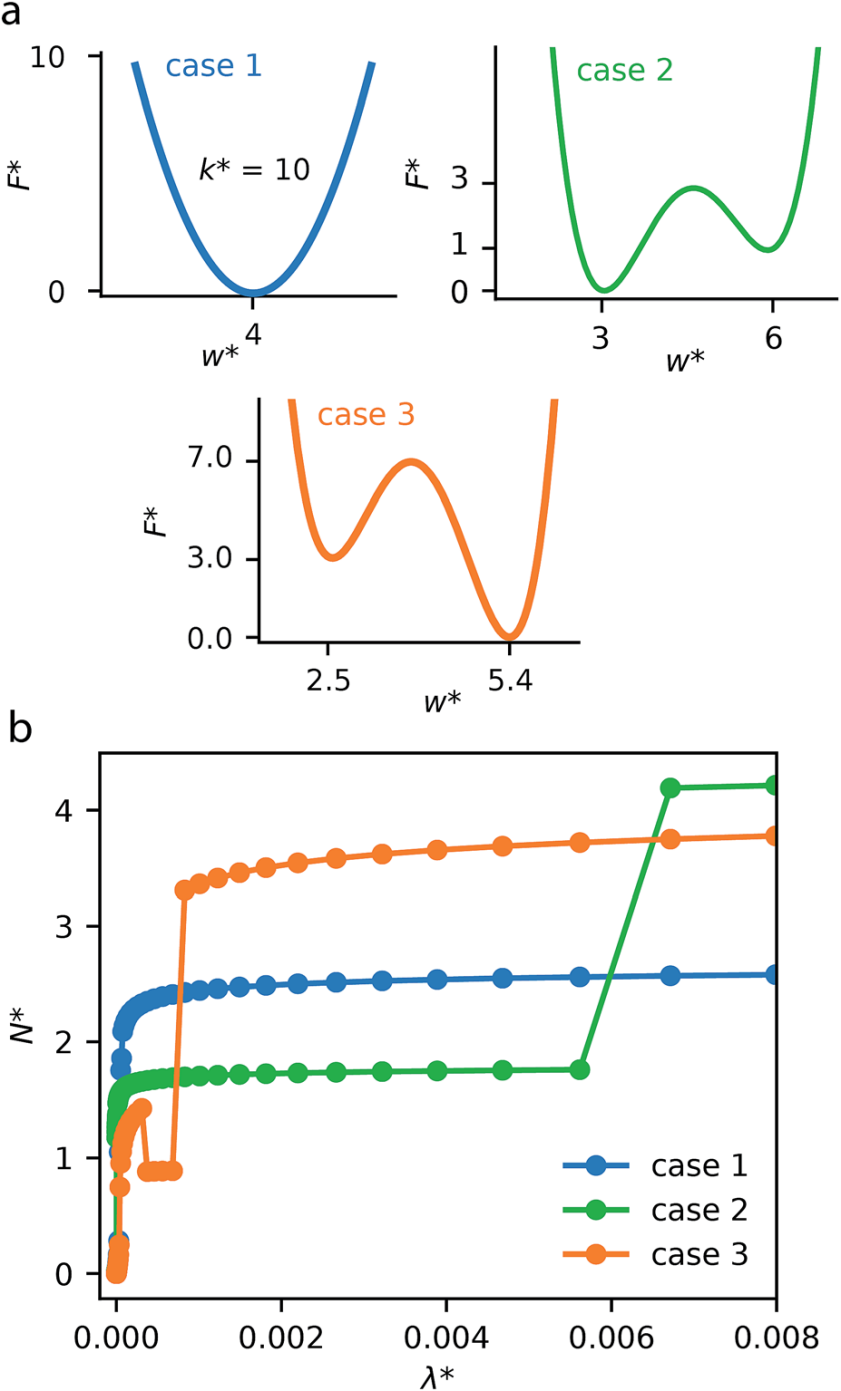
(a) Three cases of host free energy profiles and their respective critical parameters. (b) Representative gas adsorption isotherms, where N* is the adsorbed density and λ* is configurational activity, at T* = 0.80 for each of the three cases.

This generalized theoretical framework can reproduce a rich variety of gas adsorption processes. Free energy profiles, describing the potential energy surface of the slit pore width, when combined with the energetics associated with gas adsorption, allow for flexible and responsive gas adsorption mechanisms to arise. As an initial example, we demonstrate the result of three different potential profiles dictating the behavior of the slit pore system exemplified in Fig. 2.

Firstly, a tight harmonic potential with a stiffness k* = 10, case 1, produces a typical type-1 gas adsorption representative of a rigid porous material. During the process of adsorption, the osmotic potential does not display new minima, thus no changes in pore width can occur (Fig. S5†).

The free energy surface described as case 2 includes two minima with the lowest energy minimum at smaller width *E*_thin_ < *E*_wide_. At low external pressure, or configurational activity λ*, the system behaves as a rigid material at pore width at *w*_thin_. When the system reaches a characteristic gate-opening pressure the system expands to *w*_wide_, allowing for increased amount of gas uptake producing a gate-opening adsorption profile. The osmotic potential during this adsorption process (Fig. S6†) clearly shows that the smaller width minimum is further stabilized at low pressure, but at high pressure the larger width phase becomes lower in energy, allowing for an np → op transition.

Case 3 defines another free energy surface with two minima. However, for this case the lowest energy minimum corresponds to the larger width, *E*_wide_ < *E*_thin_. This potential produces a breathing-type isotherm profile where the system begins at large width, which, with increasing pressure transforms to a smaller pore state. Subsequently, at higher pressures the system will undergo a second transformation to the original pore state. These transformations op → np and np → op result from significant differences in grand potential of adsorption for different pore widths and different pressures (Fig. S7†). The reason for these differences in grand potential correspond to favorable interactions following adsorption of a single layer or multiple layers of adsorbate molecules.^31^–^33^ For example, the minima of the grand potential in pores of smaller size (below w* = 5.00) is associated with layering effects for pores completely filled with a liquid-like adsorbate.

Notably, this simulation methodology allows for the population of metastable states where a larger pore state, although less favorable than a smaller pore state, remains because the barrier between states is larger than the intrinsic fluctuation energy (reflected by the value of *Ω*_crit_). This produces the distinctive NGA step. In the ESI† we have explored different values of *Ω*_crit_, Fig. S8.† If this critical energy is zero (or very small) the adsorption isotherm always follows the local minimum of the osmotic potential producing a rigid-like isotherm. However, if *Ω*_crit_ is extremely large the system will ignore barriers between minima, following only the global minimum of the osmotic potential, once again producing no NGA but a breathing process. For NGA to occur, this value of *Ω*_crit_ is vital and represents a delicate balance between kinetics and thermodynamic driving forces. We note, a recent study was also able to describe NGA using a transfer matrix technique.^34^ However, this considered a system in equilibrium, which cannot capture metastability. From our investigations, and further work by Vanduyfhuys *et al.*,^10^ of the free energy landscapes it is found that NGA is the result of the metastability, during gas adsorption, of DUT-49.^19^

These illustrative examples highlight thermodynamic insights into responsive gas adsorption processes. For instance, the adsorption-induced deformations of the case 3 system do not simply follow the pore-width minima defined by the host free energy profile. The osmotic potential at external pressure range λ* ≈ 1 × 10^–3^ shows two distinct minima at w* = 2.175 and w* = 2.875, which correspond to favorable layering effects previously discussed (Fig. S7†). Moreover, we also observe further expansion of the pore width at higher external pressure. This is because the system can accommodate more adsorbate at higher activities. In the current model this is captured by the osmotic potential achieving lower values because of smaller values of the grand potential in fully filled larger pores, even if it comes with a penalty of increasing the slit pore width (due to the host free energy potential).

**Fig. 3:**
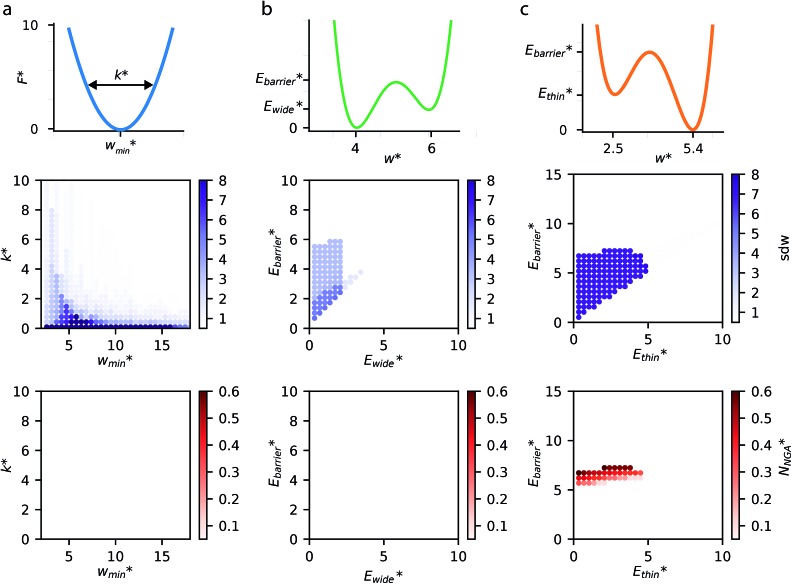
Responsive adsorption maps for three cases of free energy surfaces at T* = 0.80, (a) harmonic potential, (b) bistable potential with *E*_thin_ = 0 and (c) bistable potential with *E*_wide_ = 0. Top row: an illustration of the free energy surface and critical parameters. Middle row: amount of adsorption-induced flexibility demonstrated by the sum of differential widths (sdw). Bottom row: density of gas released (N*) by a negative gas adsorption step, if one occurs.

The above examples are representative cases that demonstrate the ability of this model to generate interesting and important gas adsorption processes. However, it is not correct to assume that any bistable system with *E*_wide_ < *E*_thin_ (case 3) to result in NGA. To explore the result of different forms of the free energy surface we conducted parameter screening resulting in responsive adsorption maps illustrated in Fig. 3. These responsive adsorption maps provide a rich understanding of the requirements of the host pore structure to result in flexibility or NGA. The amount of flexibility here is illustrated as the sum of the absolute value of differential widths (sdw) eqn (5). The magnitude of this value will be relative to the change in width during any transition and will be even larger for breathing processes, in which there are two changes in pore width.5
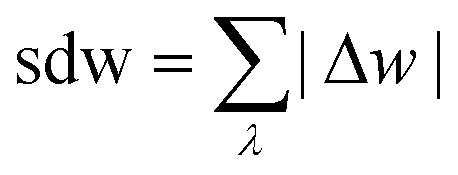



The maps were produced by generating gas adsorption isotherms for a wide grid search of conditions ranging between: 0.1 ≤ k* ≤ 10 and 
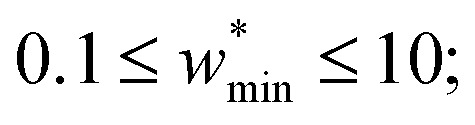
 
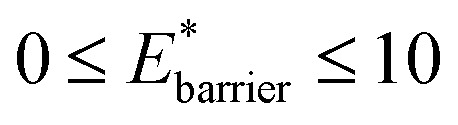
 and 
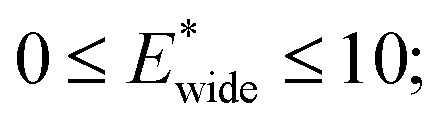
 
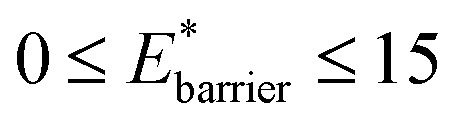
 and 
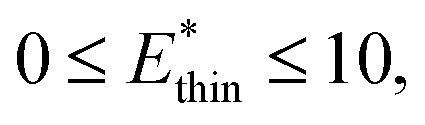
 for harmonic, bistable (gate-opening) and bistable (breathing) potentials, respectively.

For specific values of k* and 
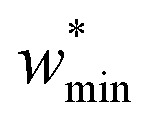
 we find that a harmonic potential can show significant flexibility. Small values of k* produce a flexible system with a broad minimum, which allows for the system of adapt to an optimal size for adsorbate multilayer formation, which as described previously is extremely favorable for slit pore systems (Fig. S4†).^35^ This produces a continuous breathing response (Fig. S9†), which has been reported for a number of porous materials.^36^,^37^ While under the conditions for metastability we imposed this system does not demonstrate NGA, however, the responsive adsorption exhibited by this potential may open new avenues for the exploration of materials that exhibit novel adsorption processes.

A bistable potential with *E*_thin_ = 0 displays three distinct responses, dependent on the critical parameters 
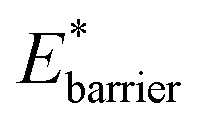
 and 
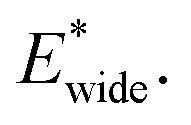
 The gate-opening transition exemplified in Fig. 2 occurs for high values of 
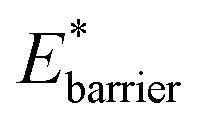
 and for 
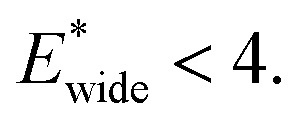
 For systems with small 
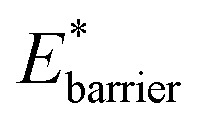
 a greater degree of flexibility is observed. These systems show an additional transition to a lower porosity and smaller *w* phase (closed pore phase, cp). From this phase, and upon greater adsorption, the system subsequently will undergo two transitions np → cp and cp → op (Fig. S10†). Notably, these three states have been observed in wine-rack framework materials.^38^

In contrast to bistable potentials with *E*_thin_ = 0, bistable potentials with *E*_wide_ = 0 display flexibility for a wider range of parameter space (*E*_thin_ and *E*_barrier_) and show consistent phase transformation as described previously (op → np → op). This is demonstrated experimentally as different systems, based on very different materials, display breathing-type adsorption processes.^39^

Fig. 3 also clearly demonstrates a bistable potential with *E*_thin_ < *E*_thin_ is crucial to produce NGA. NGA transitions occur only for a narrow subsection of this flexibility range. Only flexible systems with large barrier heights (*E*_barrier_) can permit a metastable state and produce an NGA step. Importantly, exploring the gas adsorption profiles for a wide parameter space for the other potentials did not result in NGA.

The responsive adsorption maps of Fig. 3c show only a specific choice of pore width minima (*w*_thin_ = 2.5 and *w*_wide_ = 5.4). We also investigated a series of similar responsive adsorption maps for other combinations of pore width minima as displayed in the ESI (Fig. S11–S14†). There appears to be no upper-bound for the choice of *w*_wide_ for NGA to occur, that is the op phase can have very large pore sizes (Fig. S11†). Contrastingly, there is a lower-bound for NGA processes, with respect to *w*_wide_. For a 
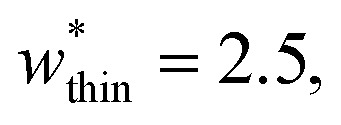
 a second minimum of 
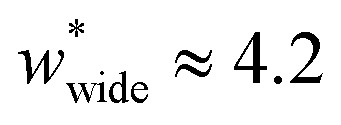
 is required to produce an NGA step (Fig. S12†). Systems with 
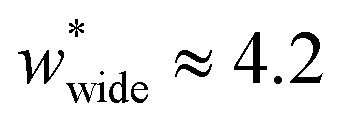
 correspond to materials that exhibit mesopore-type gas adsorption isotherms. This suggests that a mesopore → micropore transition is crucial for NGA. Moreover, we find the relative difference between the minima is important. For example, 
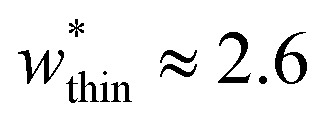
 is the largest width of a np phase for an op phase with 
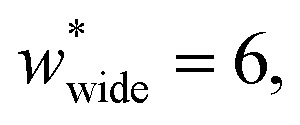
 but for a larger pore op phase the largest width np phase showing NGA is 
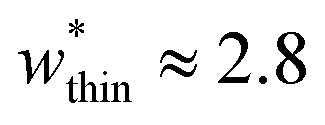
 (Fig. S13 and S14†). Furthermore, the barrier heights that show NGA are also dependent on the value of *Ω*_crit_ as displayed in Fig. S15.†

**Fig. 4:**
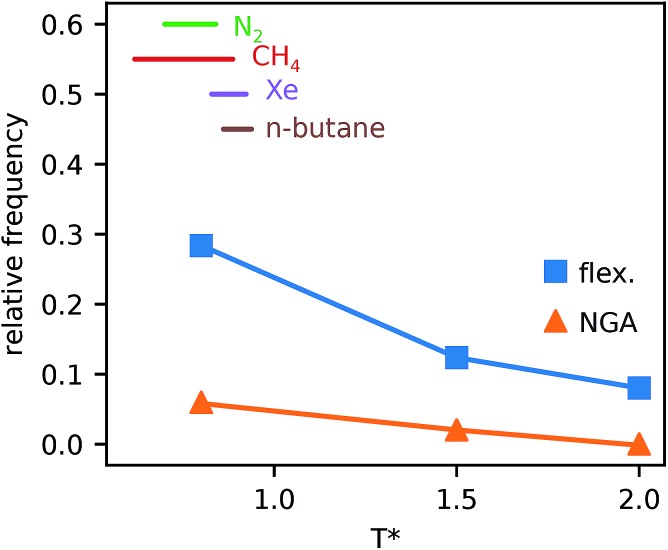
The relative frequency of systems that show changes of phase (flex.) and NGA steps (NGA) during adsorption for three temperatures compared to the temperature ranges for N_2_, CH_4_, Xe and *n*-butane, for which NGA is experimentally observed in DUT-49.

We have demonstrated the profound effects the host free energy profile can have on phase changes during adsorption including the population of metastable states. Finally, we have used the above methodology to explore the effect of increased temperature on possible NGA processes, Fig. 4.

Simulations introduced in this study were conducted at subcritical temperature (T* = 0.80), the critical temperature of the bulk Lennard-Jones fluid is T* = 1.31. However, we have also further considered temperatures close-to-critical (T* = 1.5) and supercritical fluid temperatures (T* = 2.00) and examined the effects on breathing and NGA adsorption phenomena (Fig. 4). The relative occurrence of systems that show changes in phase or NGA was determined by a thorough grid search of critical parameters 
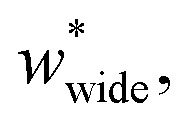
 
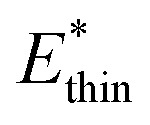
 and 
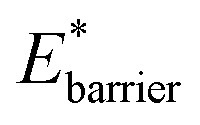
 (details of which are presented in the ESI†). At subcritical temperature, we find approximately 30% of the systems examined show changes of phase, while less than 6% show NGA. For increasing temperature, the number of these responsive systems decreases significantly to less than 2% of systems exhibiting NGA at close-to-critical temperature. At supercritical temperature, there were no systems observed that showed NGA. Responsive adsorption maps at these elevated temperatures (Fig. S16†) show that systems at higher temperature will exhibit a reduced response, only a op → np transition, in the range of configurational activities investigated. Importantly, subcritical temperature, which results in the most likely phase change and NGA behavior, corresponds to the temperature ranges for which NGA has been observed in DUT-49 during the adsorption of N_2_, CH_4_, Xe and *n*-butane.^19^,^21^,^22^

The above temperature range acts as a guide for the characterization of new materials to enable the discovery of new responsive behaviors in porous materials.

## Conclusions

We have described an approach to investigate responsive gas adsorption processes using a general simulation methodology combining the osmotic potential and classical DFT of slit pore models. This has enabled the systematic and efficient simulation and mapping of gas adsorption isotherms in soft porous materials by allowing for the pore width of the system to change size during gas adsorption according to the osmotic potential.

Employing specific free energy profiles of the host system, this model can reproduce adsorption phenomena such as gate-opening, breathing and NGA. Moreover, we have conducted comprehensive scans of the critical parameters of the host free energy profiles to produce responsive adsorption maps. These maps provide crucial insight into the specific thermodynamics responsible for phase changes during adsorption and NGA.

We subsequently used this model to highlight important design and discovery guidelines for NGA materials. Firstly, bistable systems with large energetic barriers combined with large pore sizes are required for NGA. Secondly, subcritical temperature is observed to produce more unique systems that exhibit breathing and NGA.

Our described approach enables new investigations in this burgeoning research area, allowing for both the understanding of existing material properties and the imaginative design of new responsive adsorption phenomena.

## Conflicts of interest

There are no conflicts to declare.

## Supplementary Material

Supplementary information

## References

[cit1] Davis M. E. (2002). Nature.

[cit2] Perego C., Millini R. (2013). Chem. Soc. Rev..

[cit3] Gin D. L., Noble R. D. (2011). Science.

[cit4] Horike S., Shimomura S., Kitagawa S. (2009). Nat. Chem..

[cit5] Coudert F.-X. (2015). Chem. Mater..

[cit6] Bennett T. D., Cheetham A. K., Fuchs A. H., Coudert F.-X. (2016). Nat. Chem..

[cit7] Allendorf M. D., Houk R. J. T., Andruszkiewicz L., Talin A. A., Pikarsky J., Choudhury A., Gall K. A., Hesketh P. J. (2008). J. Am. Chem. Soc..

[cit8] Horcajada P., Serre C., Maurin G., Ramsahye N. A., Balas F., Vallet-Regí M., Sebban M., Taulelle F., Férey G. (2008). J. Am. Chem. Soc..

[cit9] Mason J. A., Oktawiec J., Taylor M. K., Hudson M. R., Rodriguez J., Bachman J. E., Gonzalez M. I., Cervellino A., Guagliardi A., Brown C. M., Llewellyn P. L., Masciocchi N., Long J. R. (2015). Nature.

[cit10] Vanduyfhuys L., Rogge S. M. J., Wieme J., Vandenbrande S., Maurin G., Waroquier M., Van Speybroeck V. (2018). Nat. Commun..

[cit11] Schneemann A., Bon V., Schwedler I., Senkovska I., Kaskel S., Fischer R. A. (2014). Chem. Soc. Rev..

[cit12] Furukawa H., Cordova K. E., O'Keeffe M., Yaghi O. M. (2013). Science.

[cit13] Pera-Titus M., Farrusseng D. (2012). J. Phys. Chem. C.

[cit14] Boutin A., Springuel-Huet M.-A., Nossov A., Gédéon A., Loiseau T., Volkringer C., Férey G., Coudert F.-X., Fuchs A. (2009). Angew. Chem., Int. Ed..

[cit15] Wieme J., Lejaeghere K., Kresse G., Van Speybroeck V. (2018). Nat. Commun..

[cit16] Boutin A., Coudert F.-X., Springuel-Huet M.-A., Neimark A. V., Férey G., Fuchs A. H. (2010). J. Phys. Chem. C.

[cit17] Coudert F.-X., Boutin A., Jeffroy M., Mellot- Draznieks C., Fuchs A. H. (2011). ChemPhysChem.

[cit18] Ortiz A. U., Springuel-Huet M.-A., Coudert F.-X., Fuchs A. H., Boutin A. (2011). Langmuir.

[cit19] Krause S., Bon V., Senkovska I., Stoeck U., Wallacher D., Többens D. M., Zander S., Pillai R. S., Maurin G., Coudert F.-X., Kaskel S. (2016). Nature.

[cit20] Stoeck U., Krause S., Bon V., Senkovska I., Kaskel S. (2012). Chem. Commun..

[cit21] Krause S., Bon V., Senkovska I., Többens D. M., Wallacher D., Pillai R. S., Maurin G., Kaskel S. (2018). Nat. Commun..

[cit22] Schaber J., Krause S., Paasch S., Senkovska I., Bon V., Többens D. M., Wallacher D., Kaskel S., Brunner E. (2017). J. Phys. Chem. C.

[cit23] Evans J. D., Bocquet L., Coudert F.-X. (2016). Chem.

[cit24] Coudert F.-X., Jeffroy M., Fuchs A. H., Boutin A., Mellot-Draznieks C. (2008). J. Am. Chem. Soc..

[cit25] Ravikovitch P. I., Neimark A. V. (2006). Langmuir.

[cit26] Kowalczyk P., Ciach A., Neimark A. V. (2008). Langmuir.

[cit27] Numaguchi R., Tanaka H., Watanabe S., Miyahara M. T. (2013). J. Chem. Phys..

[cit28] Shen V. K., Siderius D. W. (2014). J. Chem. Phys..

[cit29] Sweatman M. B., Quirke N. (2002). Langmuir.

[cit30] Sweatman M. B. (2000). Mol. Phys..

[cit31] Balbuena P. B., Gubbins K. E. (1993). Langmuir.

[cit32] Lastoskie C., Gubbins K. E., Quirke N. (1993). J. Phys. Chem..

[cit33] Lastoskie C., Gubbins K. E., Quirke N. (1993). Langmuir.

[cit34] Manos G., Dunne L. (2018). Nanomaterials.

[cit35] Nguyen T. X., Bhatia S. K., Nicholson D. (2002). J. Chem. Phys..

[cit36] Choi H. J., Dincă M., Long J. R. (2008). J. Am. Chem. Soc..

[cit37] Carrington E. J., McAnally C. A., Fletcher A. J., Thompson S. P., Warren M., Brammer L. (2017). Nat. Chem..

[cit38] Nouar F., Devic T., Chevreau H., Guillou N., Gibson E., Clet G., Daturi M., Vimont A., Grenèche J. M., Breeze M. I., Walton R. I., Llewellyn P. L., Serre C. (2012). Chem. Commun..

[cit39] Férey G., Serre C. (2009). Chem. Soc. Rev..

