# Supplementary material for: Exploring the thermodynamic criteria for responsive adsorption processes
Source: Chem Sci. 2019 Apr 15;10(19):5011–7. doi: 10.1039/c9sc01299k (PMC6530534; doi:10.1039/c9sc01299k)
Supplement: Supplementary file 1 [file SC-010-C9SC01299K-s001.pdf]

Electronic Supplementary Material (ESI) for Chemical Science.
This journal is © The Royal Society of Chemistry 2019

Supporting Information for

Exploring the Thermodynamic Criteria for Responsive Adsorption Processes

Jack D. Evans[a,*], Simon Krause[a], Stefan Kaskel[a], Martin B. Sweatman[b] and Lev Sarkisov[b]

* jack.evans@tu-dresden.de

[a] Department of Inorganic Chemistry, Technische Universität Dresden, Bergstraße 66, 01062 Dresden, Germany
[b] School of Engineering, University of Edinburgh, Edinburgh EH9 3FB, United Kingdom

Detailed simulation methodology

In our model the mean field density functional theory describing the adsorption of a Lennard-Jones fluid into an ideal slit pore follows a previously reported approach.[S1]. The equation for describing the grand potential is displayed in Eq. S1, where w is the pore width, μ is the chemical potential, A is pore area, V is the external potential and f is intrinsic free energy density.

$$\Omega = A \int_0^w dz(f[\rho(z)] + \rho(z)(V(z) - \mu)) \tag{S1}$$

We partition the intristic free energy density, f, into repulsive and attractive contributions according to the Weeks-Chandler-Anderson (WCA) division of the Lennard-Jones potential $f = f_{\text{rep}} + f_{\text{att}}$, where the repulsive contribution is approximated by hard spheres. For convenience, we set the effective hard sphere diameter, d, equal to σ, the Lennard-Jones length parameter. Rosenfeld's fundamental measure functional[S2] was used for the hard sphere term and standard mean field treatment of the attractive contribution, Eqn. S2.

$$f = f_{\text{HS}}[\rho(z_1)] + \frac{\rho(z_1)}{2} \int d\mathbf{r_2}\rho(\mathbf{r_2})\Phi(r_{12}) \tag{S2}$$

where $\Phi(r)$ has the form described in Eqn. S3.

$$\Phi(r) = \begin{cases} -\varepsilon & r \leq 2^{\frac{1}{6}}\sigma \\ \Phi_{lj}(r) & r > 2^{\frac{1}{6}}\sigma \end{cases} \tag{S3}$$

The Lennard-Jones potential (Φ_{lj}) for fluid interacting with the walls of the pore is described by Eq. S4[S3] and the fluid-fluid interactions are described by a standard Lennard-Jones potential, Eq. S5. We ignore wall-wall interactions.

$$u_{\text{sf}} = 2\pi\rho_{\text{sf}}\varepsilon_{\text{sf}}\sigma_{\text{sf}}^2 \left[\frac{2}{5}\left(\frac{\sigma_{\text{sf}}}{z}\right)^{10} - \left(\frac{\sigma_{\text{sf}}}{z}\right)^4\right] \tag{S4}$$

$$u_{\text{ff}} = 4\varepsilon_{\text{ff}}\left[\left(\frac{\sigma_{\text{ff}}}{r}\right)^{12} - \left(\frac{\sigma_{\text{sf}}}{r}\right)^6\right] \tag{S5}$$

The deviation of the pore width from some equilibrium width is described by athermal Helmholtz free energy function of two general forms, Eq. S6 and Eq. S7.

$$F_{\text{host (harmonic)}} = \frac{1}{2}k(w - c)^2 \tag{S6}$$

$$F_{\text{host (bistable)}} = c_1(w - c_2)^4 - c_3(w - c_4)^2 + c_5(w - c_6) \tag{S7}$$

The mixture of quadratic and parabolic terms in Eq. S7 produce a double well function with coefficients (c_i) defining the location and depth of the minima. We consider the wall of the solid to be constructed from same particles as the adsorbate thus $\sigma_{\text{sf}} = \sigma_{\text{ff}}$ and $\varepsilon_{\text{sf}} = \varepsilon_{\text{ff}}$. However, the strength of the solid-fluid interactions can be controlled by the density of interaction sites ρ_{sf}. For a carbon wall, this density is equal to ≈38.2 nm^{-2} or ≈4.41 σ_{cc}^{-2}, where σ_{cc}=0.34 nm. Numaguchi et al argued in their studies of flexible MOFs that for stacked-layer porous coordination polymers the ρ_{sf} should be equal to ≈2.2 σ_{cc}^{-2}.[S4] As such MOFs are considered to have half the interaction site density compared to carbon walls, leading to weaker solid-fluid interaction, if $\varepsilon_{\text{sf}} = \varepsilon_{\text{ff}}$. We use this weaker interaction by setting $\rho_{\text{sf}} = 2.0$ in the present study.

Using the above parameters are library of adsorption isotherms were generated for pore widths ranging between $2.0 \leq w^* \leq 20$, with a resolution of 0.175.

The osmotic potential, Eq. S8 is then straightforwardly constructed by combining the free energy profiles of host system and the library of adsorption isotherms, as the classical density functional theory method explicitly gives the grand potential $\Omega(T, P, w)$.

$$\Omega_{\text{os}}(T, P, w) = F_{\text{host}}(w) + PV + \Omega(T, P, w) \tag{S8}$$

For a given host free energy potential (F_{host}) we can generate an adsorption isotherm by looping over configurational activity ($\lambda = \exp \frac{\mu}{kT}$), where μ is the configurational contribution to the chemical potential only, and computing the minima of the osmotic potential (Ω_{os}) for the range of pore widths (w). Thus allowing the system to change pore width for a lower energy pore according to the osmotic potential. We impose an additional kinetic criteria that the system can only change pore width if the maximum height of the osmotic potential between the current pore width and the new pore width is less than a threshold energy (Ω_{crit}) to be $6T^*$.

Gas adsorption isotherm generation is computed using a python script using the numpy, scipy and pandas packages. An example of the code used to generate the responsive isotherms can be found at the data repository of J. D. Evans at `https://github.com/jackevansadl/supp-data`. We encourage researchers to explore this methodology by combining alternative isotherm kernels and complex host free energy potentials to produce entirely new adsorption profiles.

Responsive adsorption maps (main manuscript Fig. 3) were calculated by conducting a grid search of critical parameters of the host free energy profile (Eq. S6 and Eq. S7). Specifically, 30×30 conditions were scanned between $0.1 \leq k^* \leq 10$ and $0.1 \leq w^*_{\text{min}} \leq 10$; $0 < E^*_{\text{barrier}} \leq 10$ and $0 \leq E^*_{\text{wide}} \leq 10$; $0 \leq E^*_{\text{barrier}} \leq 15$ and $0 \leq E^*_{\text{thin}} \leq 1$, for harmonic, bistable (gate-opening) and bistable (breathing) potentials, respectively. Notably, conditions where $E^*_{\text{min}} > E^*_{\text{barrier}}$ were removed from the scanning range as such criteria produced free energy profiles without two minima.

To calculate the relative frequency for three temperatures (main manuscript Fig. 4) similar conditions were scanned for the responsive adsorption maps (30 points between $0 \leq E^*_{\text{thin}} \leq 20$ and 30 points between $0 \leq E^*_{\text{barrier}} \leq 20$)but in addition w_{wide} was also scanned for 30 values between $2.6 \leq w^*_{\text{wide}} \leq 15$ this produced a total of 12180 different combinations of E^*_{thin}, E^*_{barrier} and w^*_{wide} for each temperature. From these combinations isotherms were generated and the number of isotherms which resulted in NGA and an sdw value > 0.5 (denoted flex.) were counted to give the results in Table S2 and Fig. 4 (main manuscript).

Metastability in Adsorption Processes

To understand the relationship between the free energy barriers in the system with structural transitions, critical energy fluctuations and metastability, consider the narrow pore to open pore transition as shown in the Fig. S1.

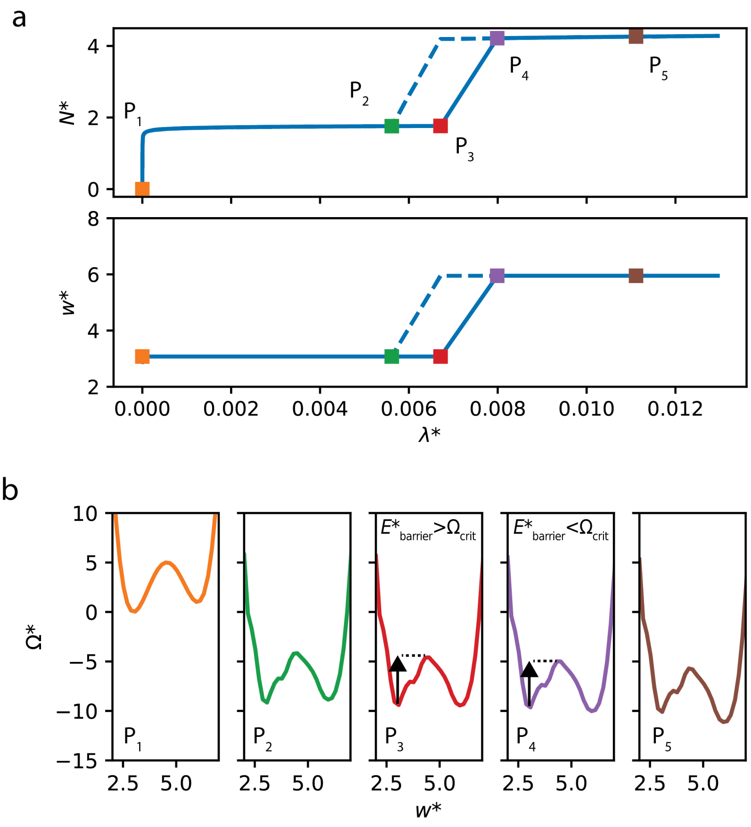

Fig. S1 Representative gas adsorption isotherm for a material which shows gate-opening, where N^* is the adsorbed density and λ^* is configurational activity. The dashed line corresponds to an equilibrium process and the solid line a processes that includes metastability. (b) Osmotic potentials at five points during gas adsorption including labeling of the critical energy barrier for a $w_{\text{thin}} \rightarrow w_{\text{wide}}$ transition.

This figure shows the behavior of volume and the amount adsorbed as a function of pressure and the osmotic potential profiles at different points on the isotherm as a function of pore widths. At the beginning (P1), there are two minima in the osmotic potential associated with the pore state at width w_{thin} and w_{wide}. The pore width w_{thin}, at this point, is lower in energy than w_{wide}, which persists at pressure P2. At P3 the w_{wide} has a lower value of $\Omega_{\text{os}}*$ so the system can expand to this lower energy state, this corresponds to the equilibrium transition. However, this transition from w_{thin} to w_{wide} is associated with an energy barrier, E_{barrier}. If this barrier is too great, the transition will not occur while the system remains in a metastable w_{thin} state. This metastable state extends until the energy barrier falls below some characteristic thermal fluctuation value (designated here as Ω_{crit}); at this point (P4) the transition can occur spontaneously. Finally at P5 the system remains in this stable state, associated with an open pore, w_{wide}.

The plausible values of the energy fluctuations have been explored by Numaguchi et al.[S4] Their argument is energy fluctuation of the system should depend on the observation time and the size of a local domain of a crystal. They employed transition state theory to formulate the rate constant for structural transition of a crystal domain (in their case, $cp \rightarrow op$ pore transition). They set the observation time to one hour and assumed that spontaneous transition occurs at the pressure for which the time constant of the spontaneous transition (=1/rate constant) is equal to the observation time. The larger is the crystal domain under consideration the deeper into

metastable region of pressure one needs to go to observe spontaneous transition within one hour. According to this analysis, at $T*$ = 0.8, the energy fluctuation of $6kT$ per unit cell corresponds to the transition domain size of 8.3×8.3 nm^2 of their model of ELM-11.

It is not straightforward to compare the numbers used in the current study to that of Numaguchi et al. however we set Ω_{crit}) to be $6kT$ and also consider the effect of this parameter.

Comparison of reduced units to a previous atomistic study

There are several studies of atomistic simulations of methane adsorption in DUT-49 at 111K.[S5,S6] Let us consider how the atomistic system approximately maps onto our reduced units, considering the critical parameters of TraPPE methane (σ_{ff} = 3.73 Åand ε_{ff} = 1.23 kJ mol^{-1}).[S7]

The structure of DUT-49 consists of one large pore ≈24 Å ($6.43\sigma_{ff}$) in diameter, 8 smaller cages of about ≈20 Å ($5.36\sigma_{ff}$) and 14 ≈10 Å cages ($2.68\sigma_{ff}$). During the collapse of the structure, under NGA conditions, the largest pore shrinks to a pore of about ≈8 Å ($2.14\sigma_{ff}$), ≈20 Å pore reduces to ≈7 Å ($1.85\sigma_{ff}$), while the smallest pore remains unchanged.

Mapping the experimental DUT-49 system on to the slit pore DFT model employed here is not a trivial task due to the difference in geometry of the systems and lack of direct correspondence between certain properties (i.e. wall density required in the slit pore model). To model the free energy profile of the host material, obtained by previous simulations shown Fig. S2, we adopt the following simplified approach. We approximate that the profile is a result of structural changes of 9 pore cages, and therefore energy change per pore cage can be easily obtained (for example, the barrier becomes 127 kJ mol^{-1}).

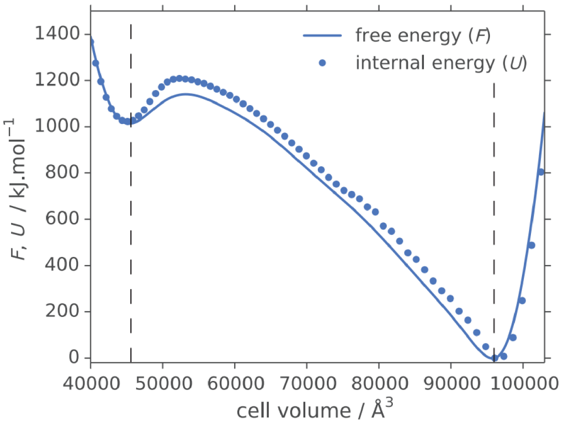

Fig. S2 Profiles of free and internal energy for DUT-49 obtained from atomistic (N, V, T) simulations as a function of unit-cell volume at 300 K. Reproduced with permission from Ref. S3.

In order to translate this energy profile into reduced units we further need to consider this energy per unit area, which is the most problematic transition. Firstly, we assume that all 9 cages are the same with pore diameter of d = 20 Å (w^*_{wide} = 5.4) that deform to 8 Å (w^*_{thin} = 2.1). The surface are of this pore can be treated as a fraction of a surface of a sphere $S = f\pi d^2$, where f = 0.2 is a fitting parameter.

Subsequently, the critical parameters of the atomistic free energy landscape (per cage E_{thin} = 112 kJ mol^{-1} and E_{barrier} = 126 kJ mol^{-1}) are reduced to E^*_{thin} = 5.0 and E^*_{barrier} = 5.6.

The resulting isotherm for this model, Fig. S3 for a slightly reduced threshold energy ($\Omega_{\text{crit}} = 5.5T^*$) displays a negative step. It would be naive to expect some quantitative accuracy here as the model does not accurately reproduce isotherms of spherical pore structures.

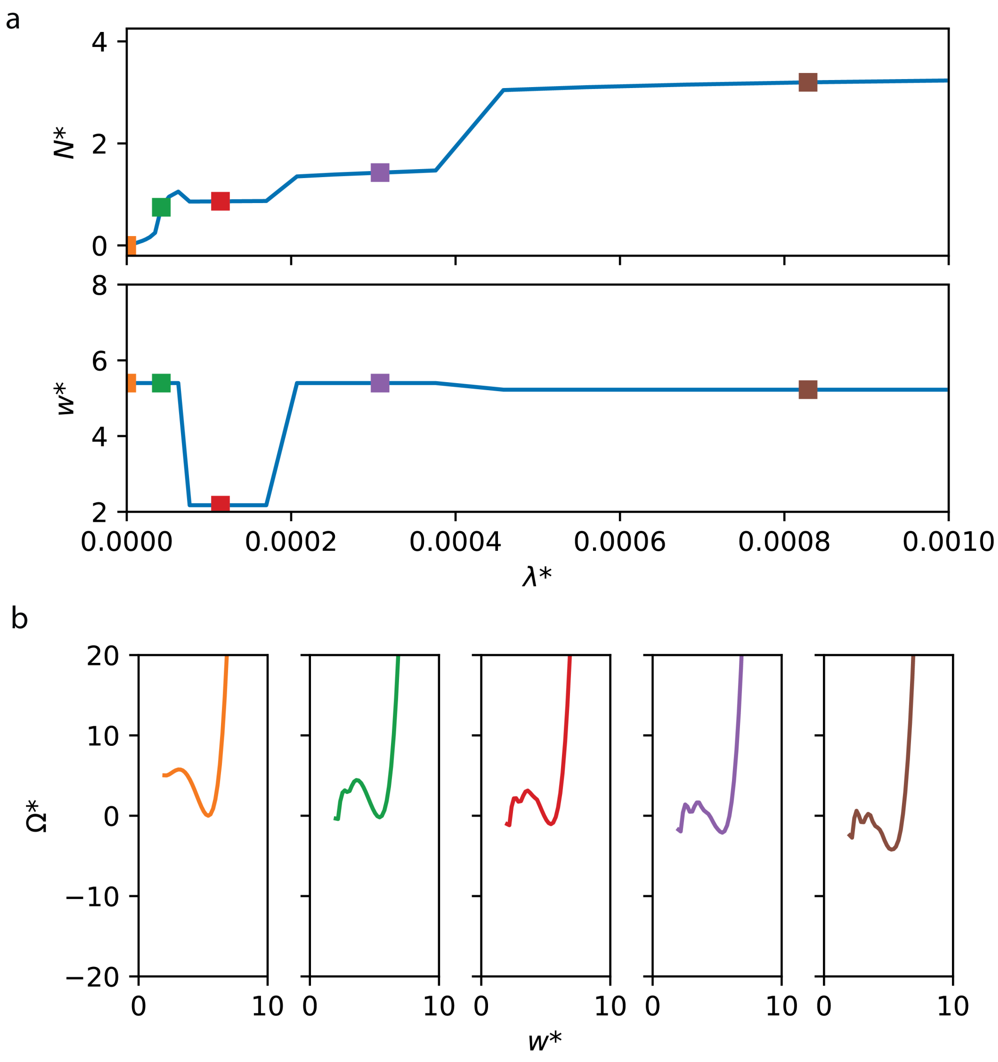

Fig. S3 (a) Representative gas adsorption isotherm for a model corresponding to DUT-49, where N^* is the adsorbed density and λ^* is configurational activity, at $T^* = 0.80$ or 118 K. (b) Osmotic potentials at five points during gas adsorption where the colors correspond to their respective points on the isotherm.

Table S1 A description of the reduced units displayed in this study.

Ω^*	=	$\frac{\Omega}{\varepsilon_{\text{ff}}\sigma_{\text{ff}}^2}$
E^*	=	$\frac{E}{\varepsilon_{\text{ff}}\sigma_{\text{ff}}^2}$
N^*	=	$\frac{N}{\sigma_{\text{ff}}^2}$
T^*	=	$\frac{kT}{\varepsilon_{\text{ff}}}$
λ^*	=	e^{μ^*}
μ^*	=	$\frac{\mu}{T^*}$
w^*	=	$\frac{w}{\sigma_{\text{ff}}}$

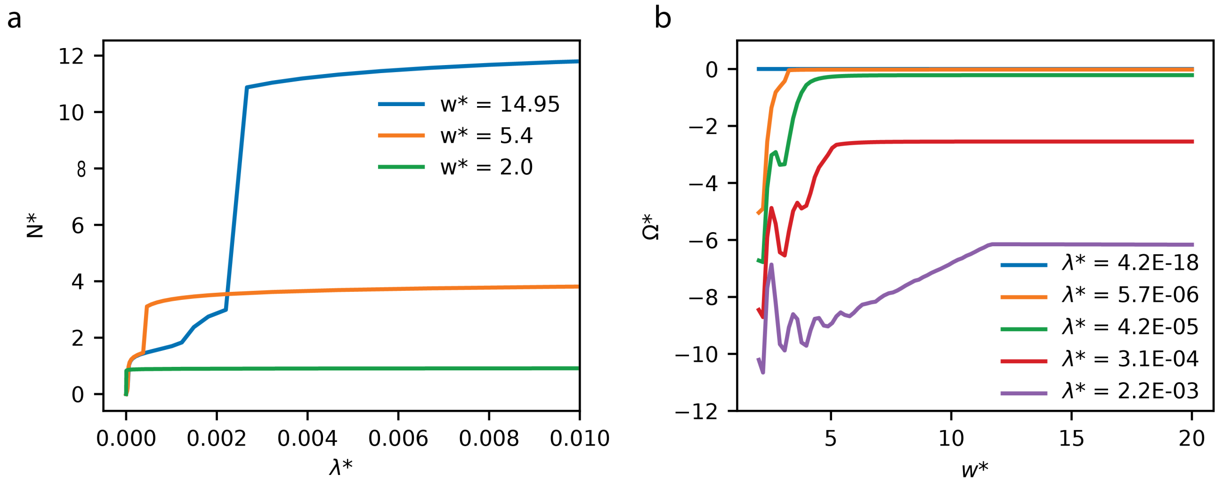

Fig. S4 Gas adsorption isotherms from the rigid slit pore systems showing (a) the amount adsorbed for increasing activity for three pore widths and (b) the dependence of grand potential (Ω) for increasing pore width for five examples of configurational activity.

Table S2 Frequency of conditions which resulted in changes of phase (flex.) or NGA steps for systems with bistable (breathing) potential at three different temperatures (12180 different systems were tested at each temperature).

T^*	flex.	NGA
0.80	3481	727
1.50	1531	266
2.00	1002	0

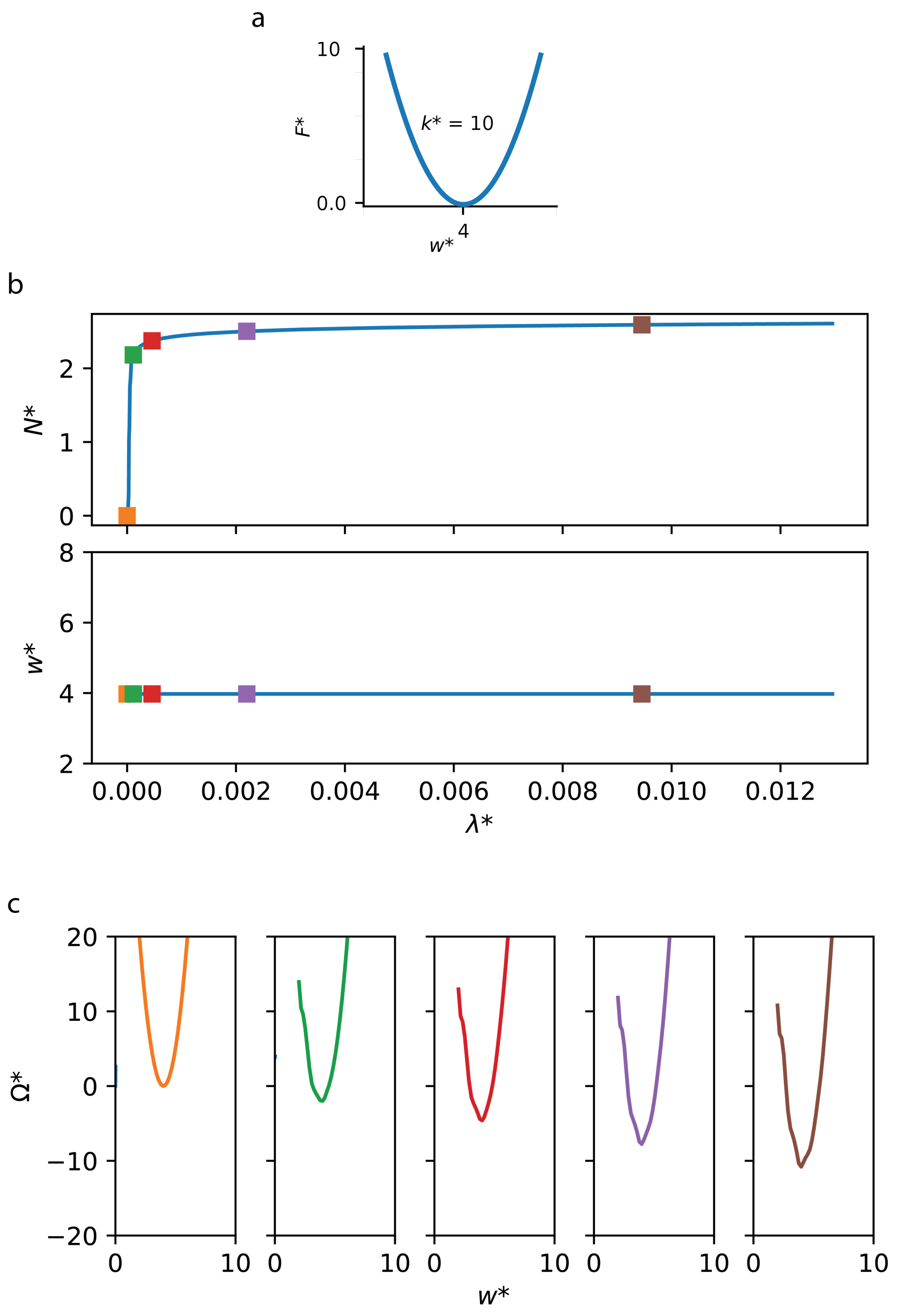

Fig. S5 (a) An illustration of a harmonic free energy profile of the host system with critical parameters labeled. (b) Representative gas adsorption isotherms, where N^* is the adsorbed density and λ^* is configurational activity, at $T^* = 0.80$. (c) Exemplary osmotic potentials at five points during gas adsorption where the colors correspond to their respective points on the isotherm.

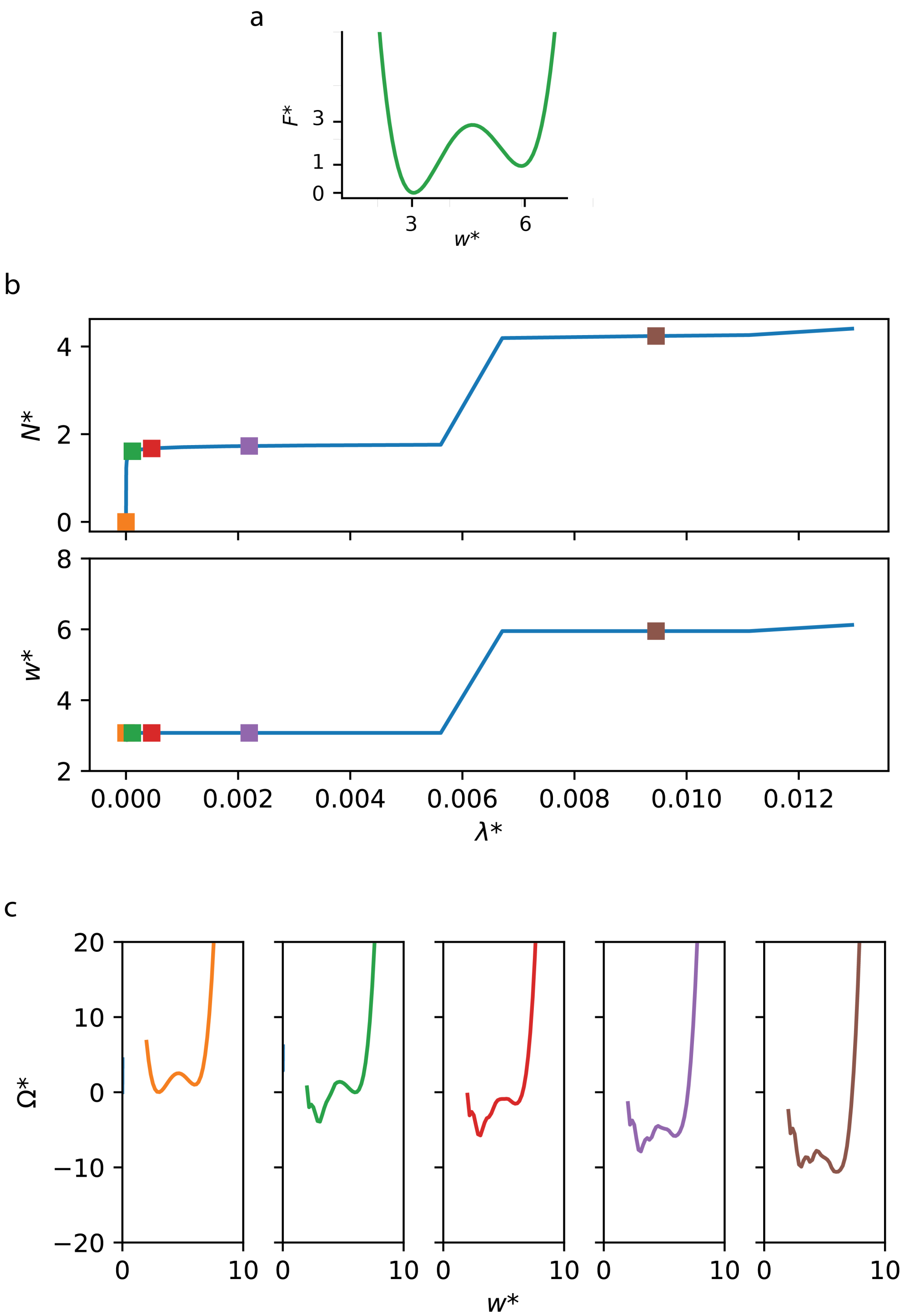

Fig. S6 (a) An illustration of a gate-opening free energy profile of the host system with critical parameters labeled. (b) Representative gas adsorption isotherms, where N^* is the adsorbed density and λ^* is configurational activity, at $T^* = 0.80$. (c) Exemplary osmotic potentials at five points during gas adsorption where the colors correspond to their respective points on the isotherm.

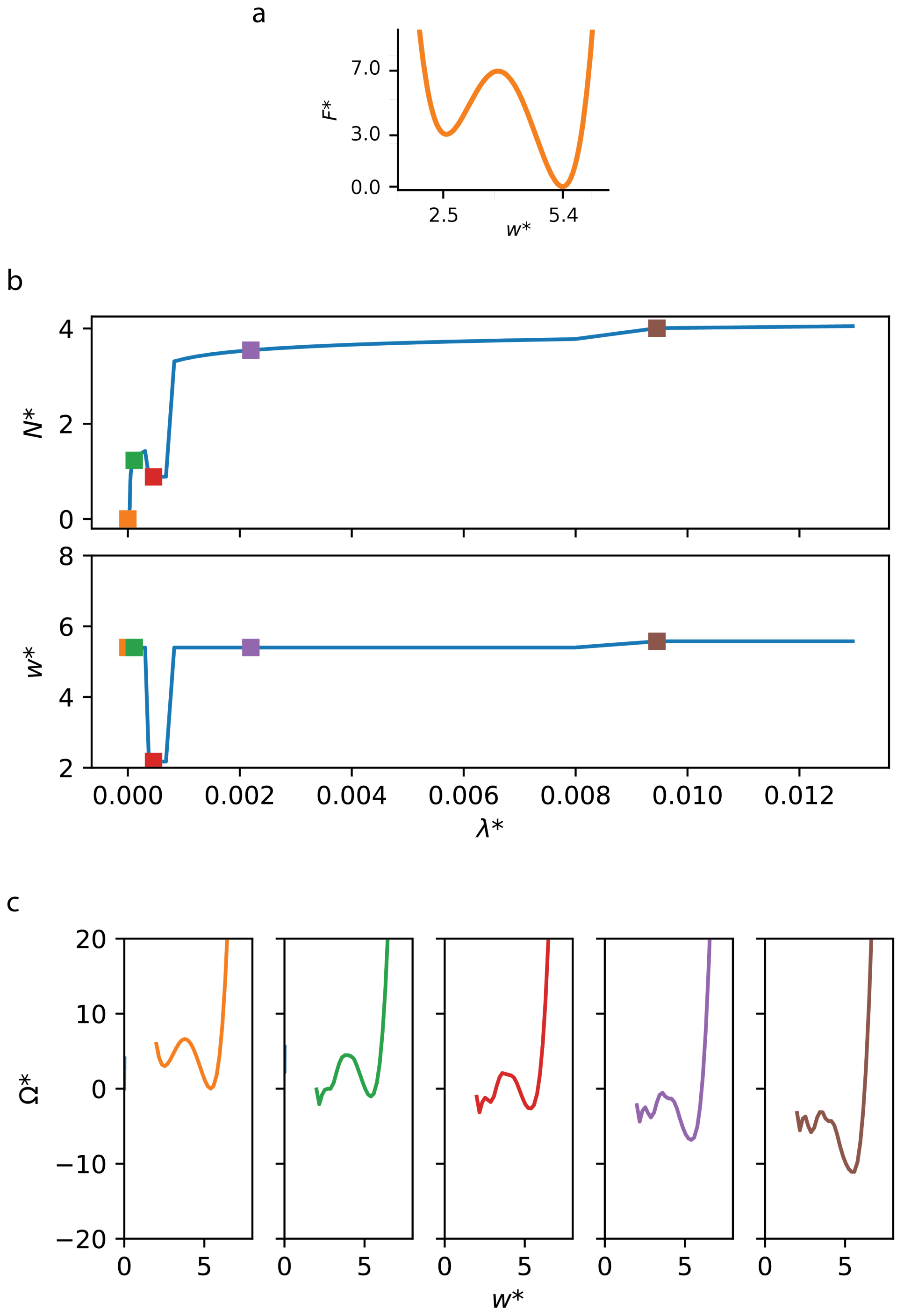

Fig. S7 (a) An illustration of a breathing free energy profile of the host system with critical parameters labeled. (b) Representative gas adsorption isotherms, where N^* is the adsorbed density and λ^* is configurational activity, at $T^* = 0.80$. (c) Exemplary osmotic potentials at five points during gas adsorption where the colors correspond to their respective points on the isotherm.

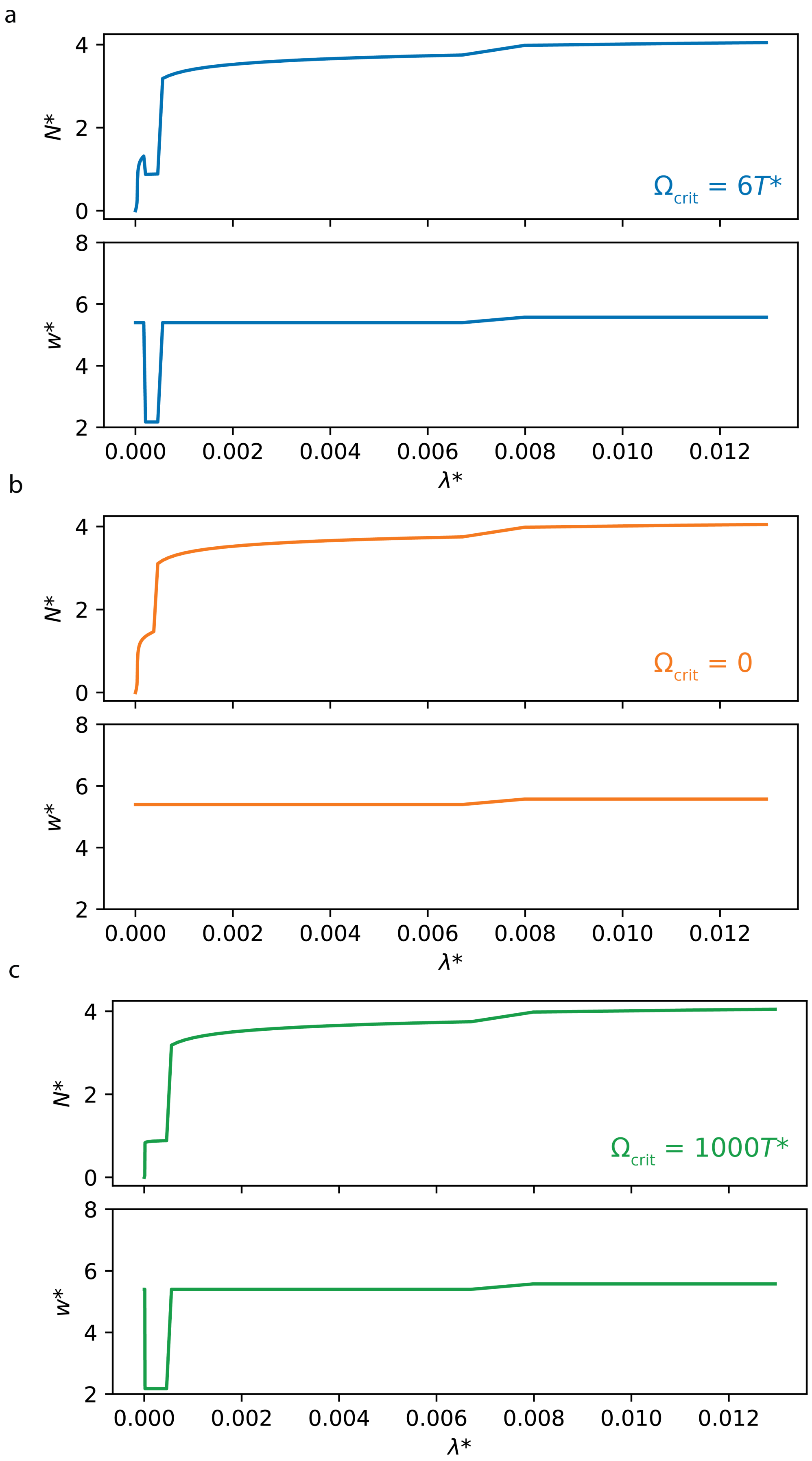

Fig. S8 The effect of different choices for Ω_{crit} for the adsorption profile of a bistable system described in Fig. S7: (a) $\Omega_{\text{crit}} = 6T^*$ (b) $\Omega_{\text{crit}} = 0$ and (c) $\Omega_{\text{crit}} = 1000T^*$

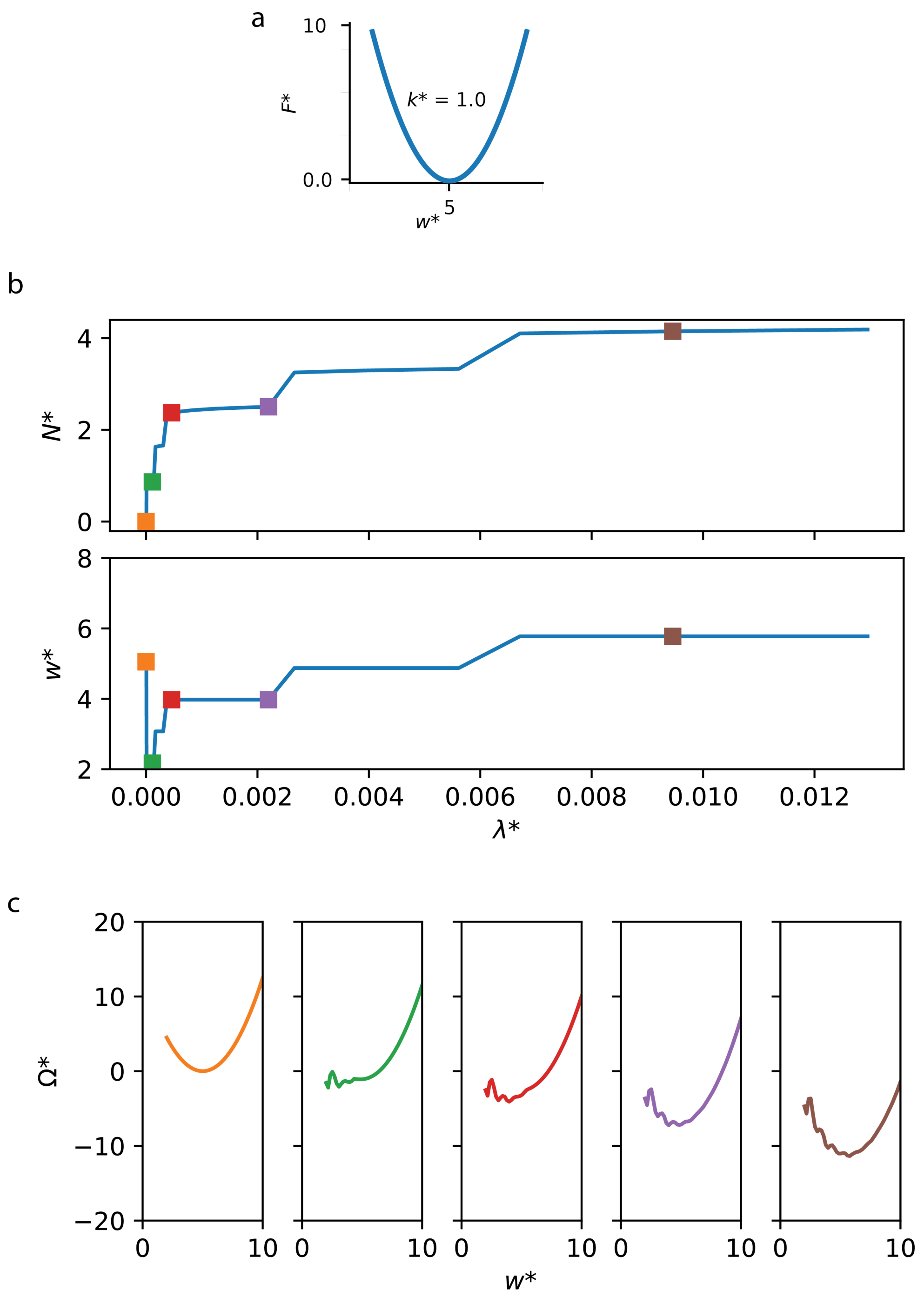

Fig. S9 (a) An illustration of a broad harmonic free energy profile of the host system with critical parameters labeled. (b) Representative gas adsorption isotherms, where N^* is the adsorbed density and λ^* is configurational activity, at $T^* = 0.80$. (c) Exemplary osmotic potentials at five points during gas adsorption where the colors correspond to their respective points on the isotherm.

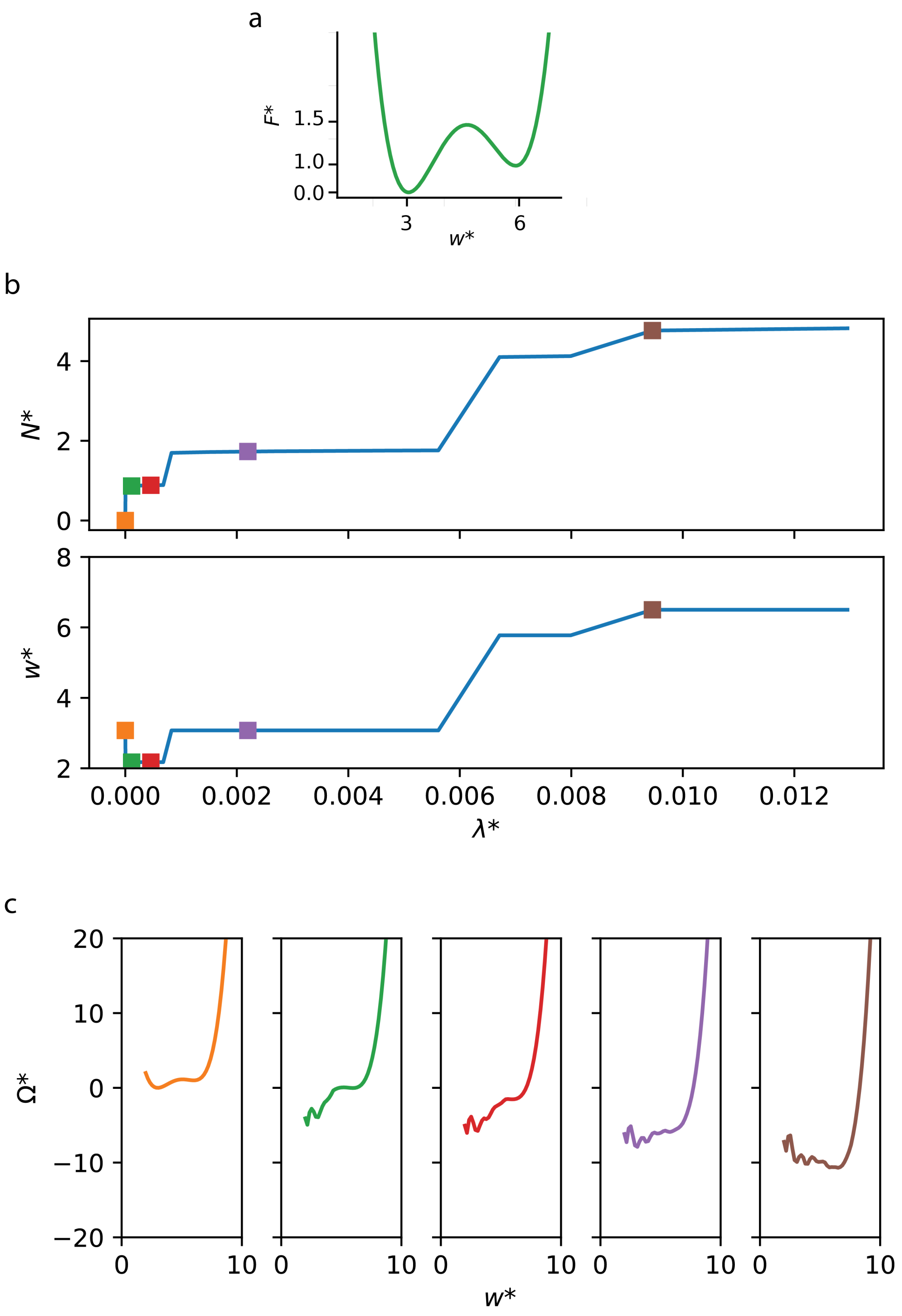

Fig. S10 (a) An illustration of a gate-opening free energy profile with small barrier height of the host system with critical parameters labeled. (b) Representative gas adsorption isotherms, where N^* is the adsorbed density and λ^* is configurational activity, at $T^* = 0.80$. (c) Exemplary osmotic potentials at five points during gas adsorption where the colors correspond to their respective points on the isotherm.

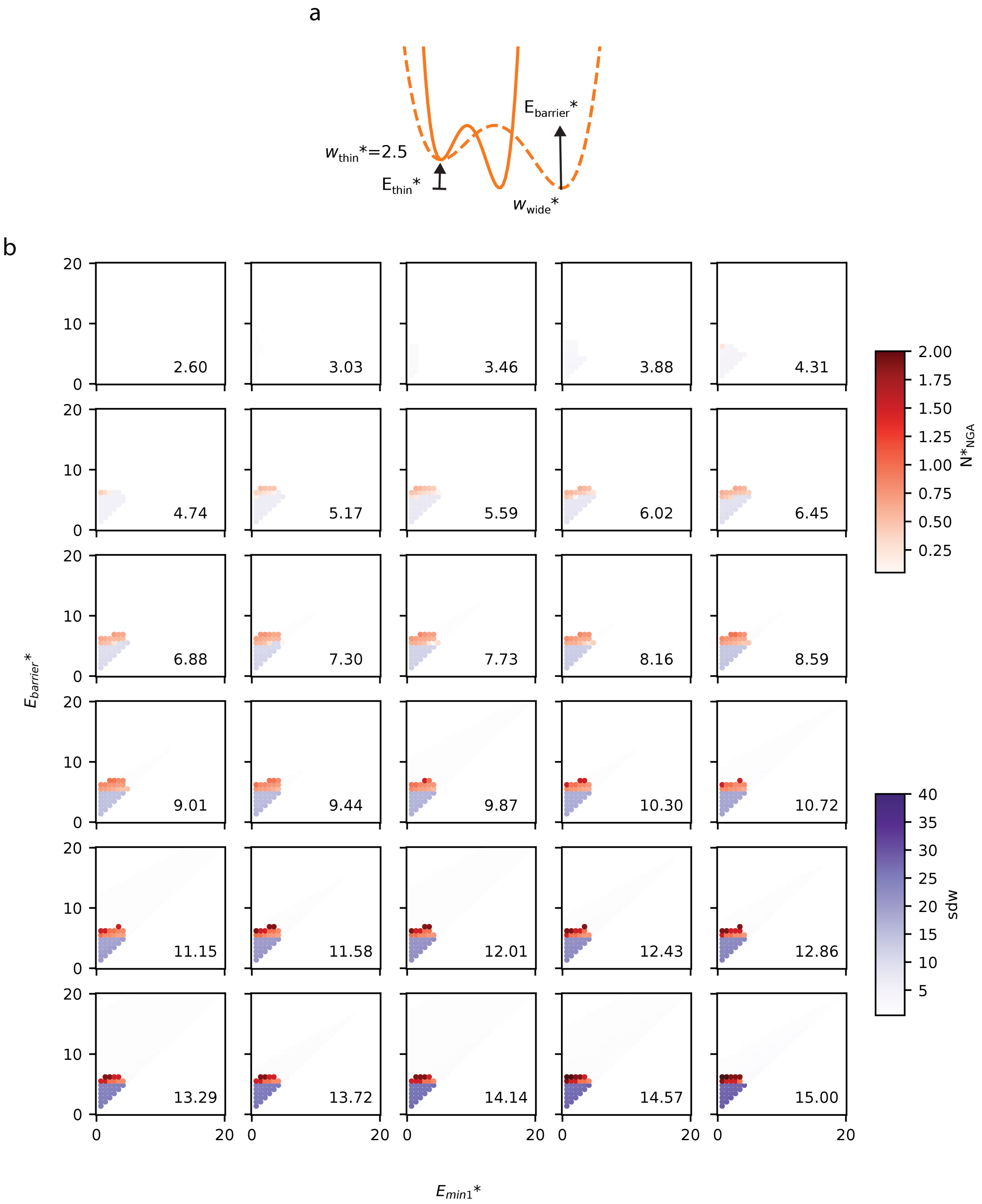

Fig. S11 (a) An illustration of free energy profile of the host system with critical parameters labeled and the effect of increasing values of w_{wide}. (b) Responsive adsorption maps for different values of w_{wide}, at $T^* = 0.80$. The amount of gas released (N^*) by a negative gas adsorption step is overlaid on the amount adsorption-induced flexibility (demonstrated by the sum of differential widths, sdw).

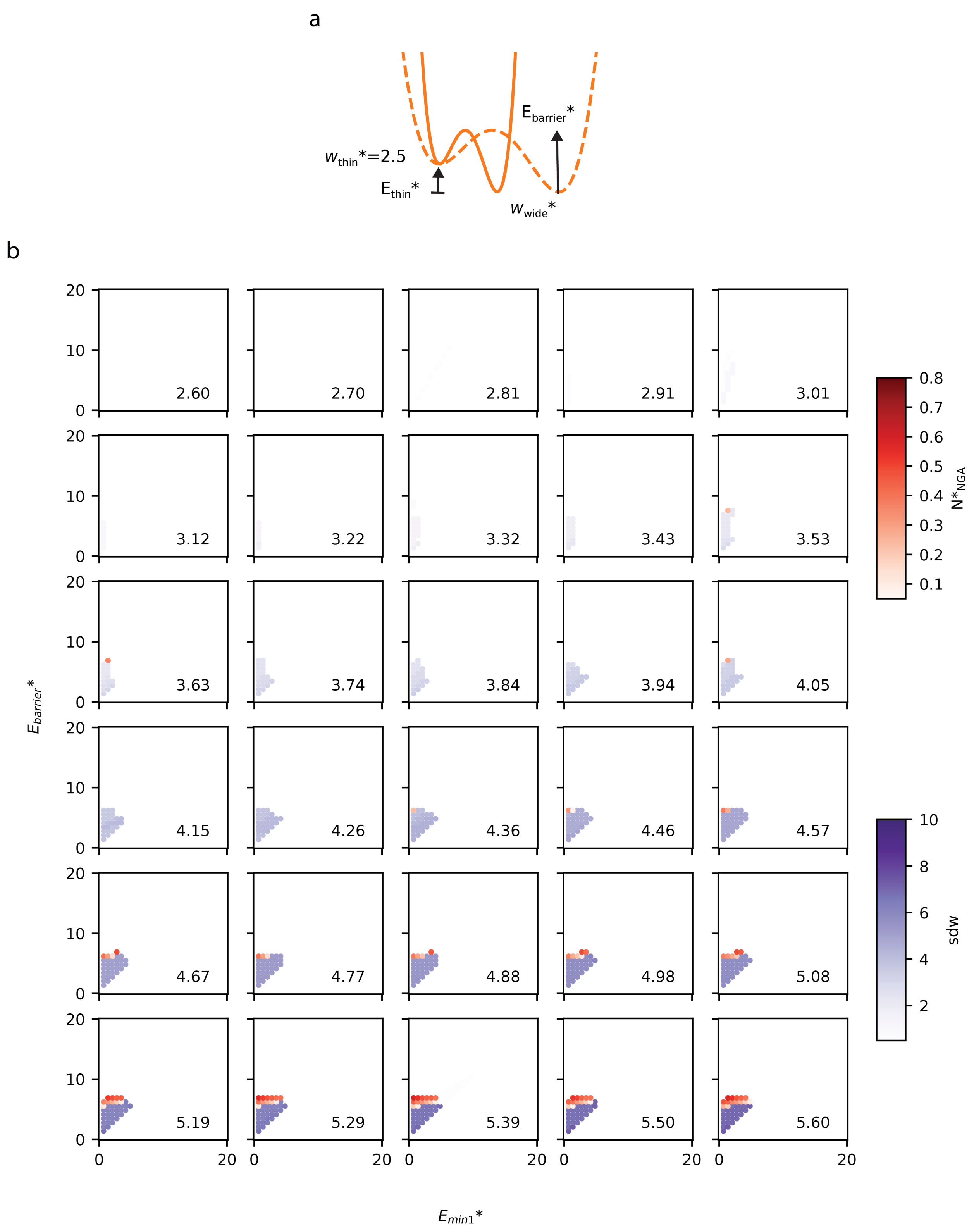

Fig. S12 (a) An illustration of free energy profile of the host system with critical parameters labeled and the effect of increasing values of w_{wide}. (b) Responsive adsorption maps for different values of w_{wide}, at $T^* = 0.80$, for smaller range. The amount of gas released (N^*) by a negative gas adsorption step is overlaid on the amount adsorption-induced flexibility (demonstrated by the sum of differential widths, sdw).

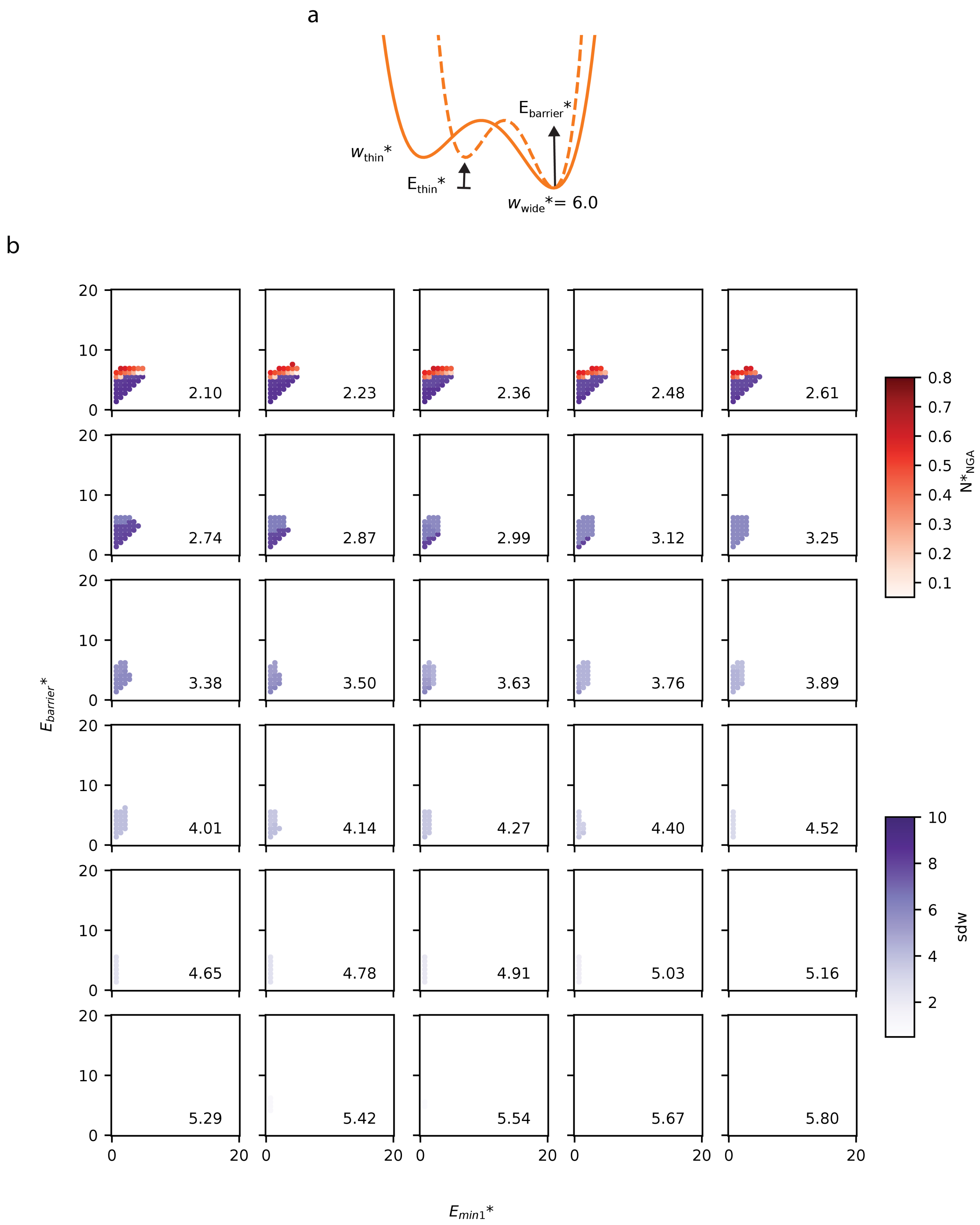

Fig. S13 (a) An illustration of free energy profile of the host system with critical parameters labeled and the effect of increasing values of w_{thin} for $w_{\text{wide}} = 6.0$. (b) Responsive adsorption maps for different values of w_{thin}, at $T^* = 0.80$. The amount of gas released (N^*) by a negative gas adsorption step is overlaid on the amount adsorption-induced flexibility (demonstrated by the sum of differential widths, sdw).

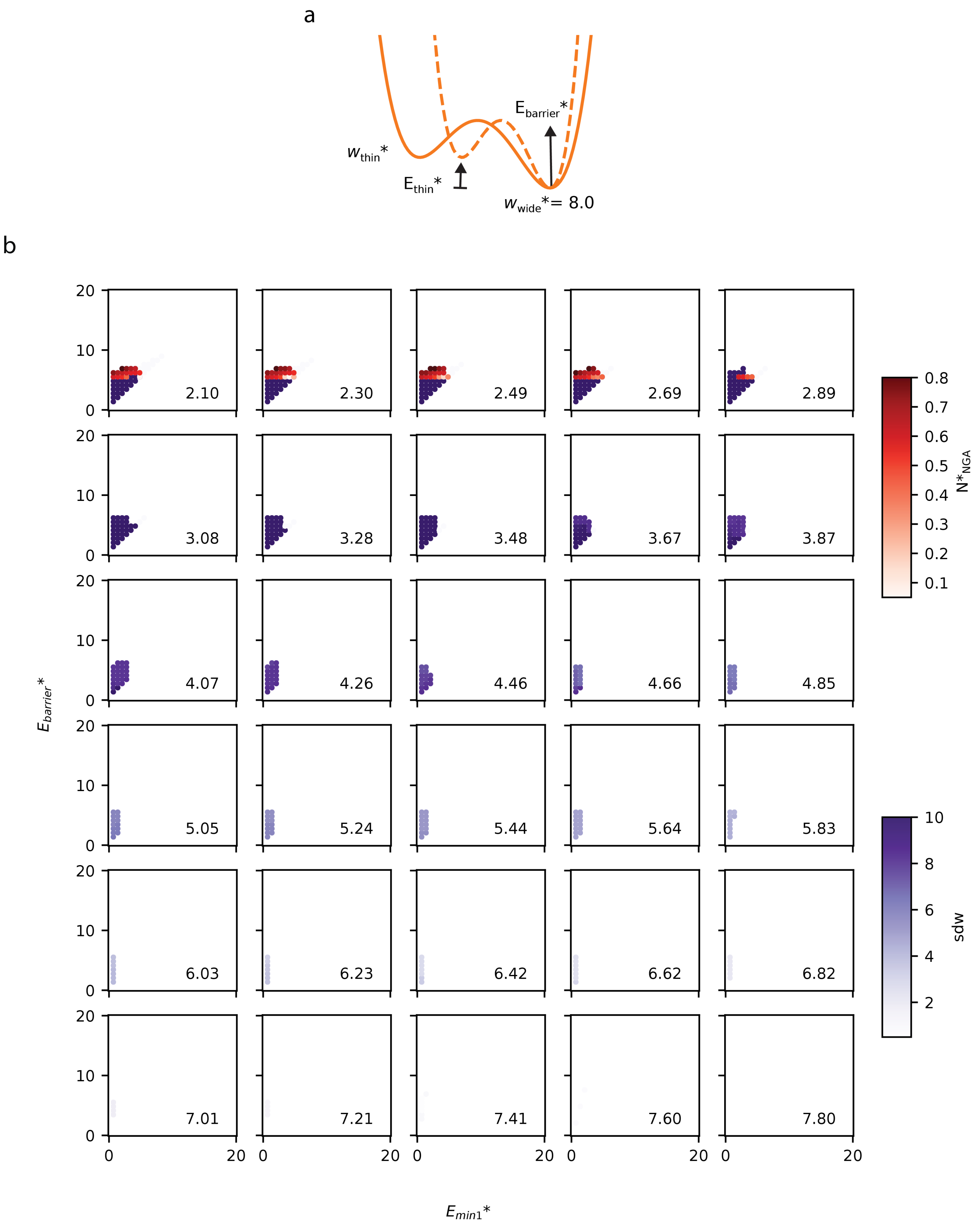

Fig. S14 (a) An illustration of free energy profile of the host system with critical parameters labeled and the effect of increasing values of w_{thin} for $w_{\text{wide}} = 8.0$. (b) Responsive adsorption maps for different values of w_{thin}, at $T^* = 0.80$. The amount of gas released (N^*) by a negative gas adsorption step is overlaid on the amount adsorption-induced flexibility (demonstrated by the sum of differential widths, sdw).

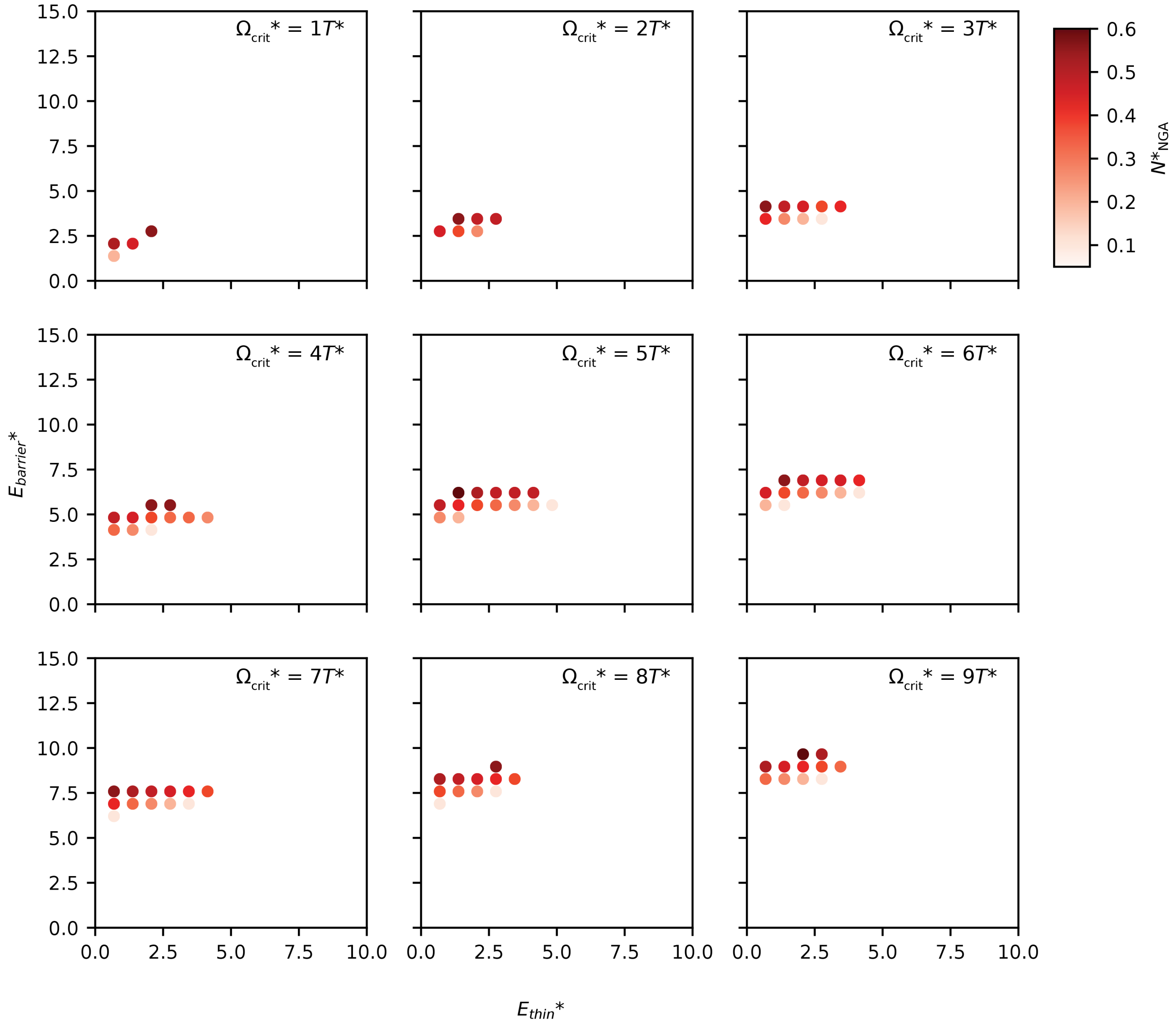

Fig. S15 Responsive adsorption maps for a system with $w_{\text{thin}} = 2.5$ and $w_{\text{wide}} = 5.4$ for different values of Ω_{crit}, at $T^* = 0.80$.

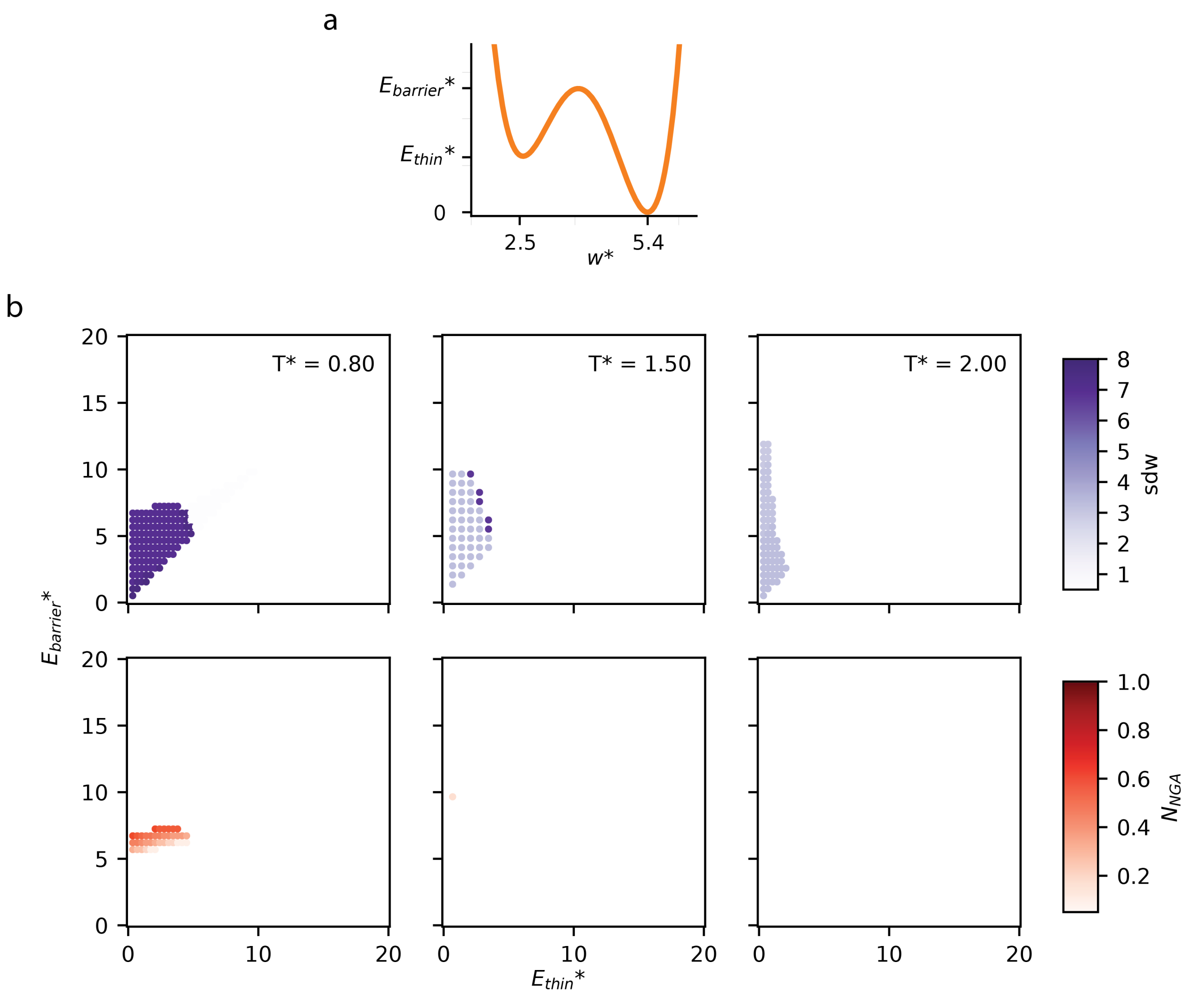

Fig. S16 (a) An illustration of free energy profile of the host system with critical parameters labeled. (b) Responsive adsorption maps for different values of T^*. (b, top row) The amount adsorption-induced flexibility demonstrated by the sum of differential widths (sdw). (b, bottom row) Density of gas released (N^*) by a negative gas adsorption step, if one occurs.

References

(S1) Sweatman, M. B. Molecular Physics 2000, 98, 573–581.

(S2) Rosenfeld, Y. Physical Review Letters 1989, 63, 980–983.

(S3) Steele, W. A. Surface Science 1973, 36, 317–352.

(S4) Numaguchi, R.; Tanaka, H.; Watanabe, S.; Miyahara, M. T. The Journal of Chemical Physics 2013, 138, 054708.

(S5) Evans, J. D.; Bocquet, L.; Coudert, F.-X. Chem 2016, 1, 873–886.

(S6) Vanduyfhuys, L.; Rogge, S. M. J.; Wieme, J.; Vandenbrande, S.; Maurin, G.; Waroquier, M.; Speybroeck, V. V. Nature Communications 2018, 9, DOI: 10.1038/s41467-017-02666-y.

(S7) Martin, M. G.; Siepmann, J. I. The Journal of Physical Chemistry B 1998, 102, 2569–2577.
